# Pronounced Seasonal Changes in the Movement Ecology of a Highly Gregarious Central-Place Forager, the African Straw-Coloured Fruit Bat (*Eidolon helvum*)

**DOI:** 10.1371/journal.pone.0138985

**Published:** 2015-10-14

**Authors:** Jakob Fahr, Michael Abedi-Lartey, Thomas Esch, Miriam Machwitz, Richard Suu-Ire, Martin Wikelski, Dina K. N. Dechmann

**Affiliations:** 1 Department of Migration and Immuno-ecology, Max Planck Institute for Ornithology (MPIO), Am Obstberg 1, 78315, Radolfzell, Germany; 2 Department of Biology, University of Konstanz, Konstanz, Germany; 3 Zoological Institute, TU Braunschweig, Braunschweig, Germany; 4 German Remote Sensing Data Center (DFD), German Aerospace Center (DLR), Oberpfaffenhofen, Germany; 5 Luxembourg Institute of Science and Technology (LIST), Belvaux, Luxembourg; 6 Wildlife Division of the Forestry Commission, Accra, Ghana; 7 University of Ghana, Legon, Accra, Ghana; Southern Illinois University, UNITED STATES

## Abstract

**Background:**

Straw-coloured fruit bats (*Eidolon helvum*) migrate over vast distances across the African continent, probably following seasonal bursts of resource availability. This causes enormous fluctuations in population size, which in turn may influence the bats’ impact on local ecosystems. We studied the movement ecology of this central-place forager with state-of-the-art GPS/acceleration loggers and concurrently monitored the seasonal fluctuation of the colony in Accra, Ghana. Habitat use on the landscape scale was assessed with remote sensing data as well as ground-truthing of foraging areas.

**Principal Findings:**

During the wet season population low (~ 4000 individuals), bats foraged locally (3.5–36.7 km) in urban areas with low tree cover. Major food sources during this period were fruits of introduced trees. Foraging distances almost tripled (24.1–87.9 km) during the dry season population peak (~ 150,000 individuals), but this was not compensated for by reduced resting periods. Dry season foraging areas were random with regard to urban footprint and tree cover, and food consisted almost exclusively of nectar and pollen of native trees.

**Conclusions and Significance:**

Our study suggests that straw-coloured fruit bats disperse seeds in the range of hundreds of meters up to dozens of kilometres, and pollinate trees for up to 88 km. Straw-coloured fruit bats forage over much larger distances compared to most other Old World fruit bats, thus providing vital ecosystem services across extensive landscapes. We recommend increased efforts aimed at maintaining *E*. *helvum* populations throughout Africa since their keystone role in various ecosystems is likely to increase due to the escalating loss of other seed dispersers as well as continued urbanization and habitat fragmentation.

## Introduction

Old World fruit bats (Chiroptera: Pteropodidae) are important seed dispersers and pollinators of a wide range of economically important tree species, thereby providing crucial ecosystem services for the maintenance and regeneration of vegetation [1–5]. Fruit bats that forage over large distances or migrate seasonally are expected to provide highly effective seed dispersal and pollination. However, wide-ranging and migratory species are especially vulnerable to anthropogenic changes because they depend on several ecosystems and are exposed to various threats when crossing ecosystems, country borders or even continents [6,7]. Given the beneficial effects of these animals as well as the potential threats they are facing, it is astonishing how little we still know about many fundamental aspects of their ecology. Hence it is crucial to understand how spatio-temporal movements and resource use by bats interact to determine the relevance of these bats for ecosystems on spatial scales of landscapes and larger.

Our study species, the straw-coloured fruit bat, *Eidolon helvum*, frequently gathers in enormous but patchily distributed colonies in many African cities such as Accra, Abidjan, Ibadan, Ife and Kampala [8–10]. In the forest zone of West Africa, large numbers of bats congregate during the dry season in colonies for roughly six months [11,12]. With the onset of the wet season, these colonies are largely abandoned when most of the bats set out for their annual migration, and only a small fraction stays behind as residents. While the timing of migration varies locally, it appears linked to climatic factors and thus ultimately to seasonal changes in resource availability [12–14].

In *E*. *helvum* as well as in other gregarious, tree-roosting Old World fruit bats, the ultimate causes for colonial aggregation are not well understood, but factors such as predator avoidance (‘selfish herd’, predator swamping) or information transfer within the colony are potential proximate explanations [14–16]. The tree roosts themselves seem unlikely to be sufficiently limited to explain such highly clumped aggregations because they will necessarily lead to intensive intraspecific competition for food resources (fruits and flowers) within the most profitable perimeter of the colony. Consequently, we should expect a density-dependent trade-off between the advantages offered by the colonies and increased resource depletion near the central place, which then requires longer travel distances to foraging areas [17].

Movement distances as well as selection of foraging areas are also key factors for effective seed dispersal and pollination, which *E*. *helvum* delivers for a large number of plants [9,14,18–22]. Indeed, straw-coloured fruit bats may account for much if not most long-distance genetic exchange of their food plants, many of which are economically important timber species [23–25].

Recent research further indicates that *E*. *helvum* is host of, and possible reservoir for, a variety of human-relevant diseases such as Lagos bat virus and paramyxoviruses [26–33]; however, actual transmission rates and pathways remain unknown. Urban bat colonies are in close contact with humans, which is exacerbated by massive hunting and consumption of bats as bushmeat in many parts of West and Central Africa [34,35]. Revealing movement patterns of these bats is thus an essential component to understand transmission of diseases for which they might be a reservoir.

We studied the foraging ecology of straw-coloured fruit bats and concurrently monitored the seasonal fluctuations of the colony in Accra, Ghana. Specifically, we hypothesized that travel distance to, and size of, foraging areas should increase during peak times of colony size when intraspecific competition for food resources should be highest. We further expected to find trade-offs between distances travelled and activity budgets. Habitat use should be more selective during low colony size if individual bats have more options to choose foraging areas under less crowded conditions. Finally, we predicted that central place foragers would commute greater distances when controlling for the predicted positive relationship between body size and travel distances of Old World fruit bats. We tested these hypotheses with high-resolution GPS and acceleration telemetry combined with remote sensing data, ground-truthing of utilized food resources as well as population monitoring of the focal colony.

## Methods

### Ethics statement

Our study adhered to the guidelines of the American Society of Mammalogists for the use of wild mammals in research [36]. Research was carried out under permits issued by the Wildlife Division of the Forestry Commission (P.O. Box M239, Accra, Ghana; permits FCWD/GH-01 24/08/09 and 02/02/11). Permit to work within the compound of the 37 Military Hospital was granted by Colonel Samuel Bel-Nono, director of the Veterinary Services, Ghana Armed Forces Medical Directorate. According to Ghanaian laws no further ethical approval by a committee was required for this study.

### Study site and animals

The study was conducted during two field seasons with straw-coloured fruit bats, *Eidolon helvum*, from a colony on the grounds of the 37 Military Hospital in Accra, Ghana (5°35'11''N, 0°11'02''W). The first bout of fieldwork took place in August 2009 during the wet season while the second bout of fieldwork was conducted in February 2011 during the dry season.

We caught bats when they returned from foraging in the early morning with canopy mist nets [37] and a 10 m high macro net [38], and kept them individually in soft cloth bags until processing. Capture upon return in the morning ensured that the animals had fed before handling. We weighed all bats with Pesola spring balances and selected 30 adult males (10 in 2009, 20 in 2011; mean mass: 277 ± 26 g). We attached GPS loggers (e-obs, Munich, Germany, mass: 19.5 g in 2009, 20 g and 24 g in 2011; see also S1 Table) by clipping the dorsal fur below the shoulder blades and gluing on the loggers with Sauer Hautkleber (Manfred Sauer GmbH, Lobbach, Germany). Previous experience with other bat species showed that loggers are shed after a maximum of two weeks. Logger mass amounted to 6.8–8.8% of the bats’ body mass, which is slightly above the recommended mass [39], but within the 5–10% range recommended through a meta-analysis of tracking studies [40]. All animals were then hand fed *ad libitum* with banana. After release near the capture site all bats flew off without any apparent difficulty. Bats were named after the serial number of the logger they carried (Table 1).

**Table 1:** Size of core areas (50% UD) and foraging areas (home ranges; 90% UD, 95% UD) of bats tracked in wet vs. dry season. Kernel density estimation calculated with fixed smoothing and bandwidth (*h*) equalling mean distance between successive foraging points (wet: 124 m, dry: 187 m). LoCoH: local convex hulls, with *k* referring to the number of nearest neighbours used for constructing local hulls, and n° of points included at the respective UD bin.

	Bat #	total n° points	50% UD				90% UD				95% UD			
			kernel	LoCoH			kernel	LoCoH			kernel	LoCoH		
			area [ha]	area [ha]	k	n° points	area [ha]	area [ha]	k	n° points	area [ha]	area [ha]	k	n° points
Wet	1079	141	15.54	0.09	10	71	60.02	2.21	10	130	79.21	5.43	10	141
	1080	101	11.73	0.08	9	51	39.11	0.52	9	93	50.38	0.71	9	97
	1081	121	7.41	0.03	10	65	24.48	0.16	10	109	31.81	0.32	10	117
	1082	47	21.33	0.13	6	24	110.28	3.74	6	43	149.23	156.25	6	47
	1084	119	15.24	0.11	9	60	53.94	0.73	9	108	70.16	1.06	9	119
	1086	48	12.32	0.25	7	27	71.93	10.98	7	44	99.68	56.69	7	48
	1088	202	10.72	0.04	11	101	32.84	0.72	11	182	41.85	2.26	11	200
	Mean		13.47	0.11			56.09	2.72			74.62	31.82		
	Median		12.32	0.09			53.94	0.73			70.16	2.26		
Dry	1607	186	82.25	0.22	12	94	461.53	93.66	12	173	686.11	642.41	12	178
	1608	27	42.08	0.34	5	17	167.19	2.62	5	25	220.46	630.17	5	27
	1610	30	60.40	2.61	5	15	272.19	74.81	5	30	359.24	74.81	5	30
	1612	25	27.21	0.02	6	13	99.53	0.14	6	24	130.69	0.14	6	24
	1613	39	25.52	0.06	4	22	112.67	2.48	4	36	152.82	4.41	4	39
	1615	29	20.53	0.04	5	15	138.51	149.92	5	27	199.11	412.96	5	29
	1616	75	40.18	0.06	9	44	192.44	0.85	9	70	281.95	4.86	9	73
	1620	90	42.28	0.13	8	45	176.18	3.89	8	85	237.93	4.44	8	86
	1626	164	16.11	0.02	8	82	66.38	0.38	8	148	94.80	1.42	8	156
	Mean		39.62	0.39			187.40	36.53			262.57	197.29		
	Median		40.18	0.06			167.19	2.62			220.46	4.86		

### Tracking loggers

The loggers are capable of recording several types of data (GPS bearing, flight speed and heading, altitude, and 3-axial acceleration data), and are flexibly programmable regarding sampling rates as well as onset and intermission of data collection. In addition, they contain a pinger which produces a signal equivalent to that of a radio transmitter, and can be turned on at specified times to facilitate localizing the animals and approach them to within downloading distance of the UHF radio link. We programmed the loggers according to data collecting regimes consecutively called cohorts 1–3 (see below and S1 Table). The following parameters were the same for all loggers deployed in 2009: delayed start at 06:00 on the morning following release; acceleration data: data collection 15 s/min at a byte count of 1188 (56.23 Hz) on three axes (x = left-right, y = back-forward, z = up-down) during the entire data collection periods (day and night); GPS: off from 06:00–18:00 each day. The only difference was that cohort 1 (#1079–1083) was programmed to collect GPS fixes at a regular interval of 600 s during GPS on-times. Cohort 2 (#1084–1088) collected GPS fixes once every 900 s until an animal was moving at a speed of 5 m/s or more. At this point cohport 2 switched to a collecting interval of 300 s. Loggers deployed in 2011 (cohort 3) had the same settings but started data collection immediately after release of the animals. In addition, they collected GPS fixes once every 1800 s until the animal started flying. Then GPS fixes were collected every 300 s (acceleration informed, see [41]).

For data download we walked at least once during daytime through the colony with a base station connected to a directional high-gain antenna (e-obs). All data were subsequently uploaded to Movebank (<www.movebank.org>), a global repository of animal movement data.

### Classification of acceleration data into discrete behaviours

To calibrate the acceleration data, we attached one logger to a captive *E*. *helvum* in a large flight cage of the Accra Zoo and observed the bat’s behaviour for several hours. The pinger signal of the logger briefly speeds up before the 15 s-collection bout of the accelerometer, which allowed us to record the exact behaviour of the animal during this time. Acceleration data were then plotted with a visualization tool (Movebank acceleration viewer; <http://www.3dyne.com/movebank-acceleration-viewer>). We classified acceleration data into behaviours based on discrete patterns (see S1 Fig for examples). We distinguished between the categories “resting” (sleeping or otherwise immobile), “moving” (active but not flying), “flying” (bursts that were entirely composed of flying activity, which represented commuting flights between day roost and food trees or between food trees), “starting” (bursts where the animal started flying at some point during the 15 s), “landing” (where the animal was initially flying and then landed during the 15 s) and “short flight” (which started and ended within the 15 s and consisted just of a few wing beats). These last three categories of flights lasting one minute or less were summarized into “foraging flights” (i.e., short flights within food trees or between food trees and feeding perches). Our classification of flight data into commuting or foraging flights was verified by visual inspection of the GPS locations in Google Earth. More fine-grained classification of behaviours (see S1 Fig) would be possible, but were beyond the scope of our study.

### Acceleration data analysis

We used data from full 24 h-cycles allowing missing 15 mins at each end and always starting at 18:00 hours UTC (= Ghana local time). We defined the beginning of “day” to be at 6:00 hours and the beginning of “night” at 18:00 hours, roughly corresponding to sunrise and sunset. We then calculated the percentage of time spent resting, moving, commuting and foraging separately per night and per day (commuting and foraging did not occur during the day). We tested for differences in the acceleration data with Mann-Whitney-U tests in InStat Version 3. Values are reported as means ± standard deviation unless otherwise noted. Significance level was 0.05 and all tests were two-tailed.

### Spatial data analysis

GPS-points were classified into three behavioural categories: “roost” (all points in the immediate vicinity of the colony), “commute” (points connecting “roost” and “forage”, i.e., when bats left and returned to the colony, and points connecting discrete foraging areas), and “forage” (clustered points around foraging trees). Points were initially classified into these categories based on their spatial context. We subsequently checked our classification with the acceleration data where “commute” included a consecutive row of acceleration bursts classified as “flying” (see above) either between the colony and the first or last foraging area visited in a night, or between discrete foraging areas. GPS-points classified as “forage” included all behavioural categories of the acceleration analysis.

Spatial data were analysed with ArcView 3.2a (ESRI, California, USA) in UTM coordinates (UTM zone 30N, WGS84). Utilization distributions (UDs) were calculated with two approaches (partly for comparability with other studies): kernel densities and local convex hulls (LoCoH). Kernel densities were estimated for each animal from foraging points with bivariate normal kernels and fixed smoothing. The smoothing factor *h* (bandwidth) was calculated as the mean distance between successive foraging points of all individuals within a tracking season [42]. Kernel density estimations were computed with the “Home Range Extension for ArcView” (HRE, ver. 1.1c, [43]) in percent volume, in 10%-contour steps plus the 95% contour, and with a 550*550 m grid. X- and Y-bandwidths were not standardized. We further estimated UDs with local convex hulls (ArcView extension LoCoH, ver. 2.1). The number of local neighbours (*k*) used for constructing local hulls was first evaluated by calculating a range of LoCoHs with *k* set to 3–15. We subsequently checked graphs where area was plotted as a function of *k* to identify jumps in area size. We visually inspected the gap-filling properties of the resulting local hulls around these area jumps and identified an optimized *k* for each individual by selecting 1) a value that avoided spurious holes in the core foraging area and 2) which was less than or equal to the square root of the number of foraging points [44,45]. LoCoHs were then calculated with the selected *k* in 5%-density steps up to 100%. We defined core areas as those enclosed by a 50% UD and present foraging areas as both 90% and 95% UD isopleths (see Börger et al. [46], who recommend the 90% rather than the commonly used 95% isopleth).

Cumulative distance flown per night and individual was calculated as straight lines connecting all points from 18:00 hours until 6:00 hours. Maximum foraging distance was calculated for each individual from the midpoint of the colony to the most distant GPS-point classified as foraging.

### Habitat use

We used two land cover data sets derived from remote sensing to assess habitat use of *E*. *helvum*. Foraging in relation to tree cover was assessed with a regional MODIS-based data set corresponding to percent tree cover (“fractional cover”) and with a spatial resolution of 232 m (derived from MOD13Q1 and spanning from 15°25'N, 5°52'W to 4°40'N, 2°32'E, [47,48]). Fruit and flower resources of *E*. *helvum* are woody plants, hence fractional tree cover should correspond to the density of woody plants within a grid cell potentially available to foraging bats. Use of urban habitat was evaluated with data of the radar satellite TerraSAR-X [49,50]. This data set is a binary classification of built-up areas (grid value “1”) and areas without buildings (grid value “0”). The original data with a spatial grain of 4 m were additionally aggregated to 100 m and 232 m resolutions with the maximum value rule, i.e., larger grid cells containing at least one smaller grid cell classified as built-up were aggregated to “built-up”. We used this procedure to assess the bats’ use of urban habitat with the reasoning that even areas around a single building would have a human footprint.

Grid values of fractional tree cover (%) and built-up (0–1) were extracted for each GPS-point classified as “forage” (see above: Spatial data analysis). These values were compared to random points created within a circular buffer of 88 km around the colony, corresponding to the maximum foraging distance of the dry season. Excluded from this spatial buffer were inland water bodies and ocean as defined by the SWBD data set (SRTM Water Body Data, Shuttle Radar Topography Mission). Water bodies and areas beyond coastline were set to “no data” and excluded for the creation of random points. Fractional tree cover ranged between 8.1 and 80.1% within the extent of the 88 km-radius. Random points (10,000) were generated with Animal Movement (version 2.04, [51]) and a distance-weighted function, i.e. with point density decreasing proportional to the distance from the colony [52].

To balance the contribution of individuals to the habitat utilization analysis, we chose the lowest number of GPS locations of any individual in each season (wet: n = 51 foraging locations; dry: n = 25), and randomly sampled all individuals with a higher number of locations, thereby reducing them to the same number of bearings [53]. We also calculated the mean land cover value for each individual and then tested for seasonal differences in habitat use.

### Feeding behaviour

We visited most of the foraging areas of tracked bats by homing in on their GPS-coordinates. The high accuracy of the data allowed us to distinguish between trees used for gathering food and those used to consume food and/or rest (S2 Table). Food trees were identified by bearing either fruits or flowers during our visits while trees used as night roosts lacked food resources on the tree, but frequently showed feeding signs such as partially consumed fruits or ejecta pellets under the tree. Visited tree species were either identified in the field or by providing botanists with pictures of the food trees (see Acknowledgments).

### Colony size estimation

We built on previous efforts monitoring the size of the bat colony in Accra (see [54]). From January 2009 through January 2012, we strived to conduct visual counts on a monthly basis. For this, a single, trained observer walked through the colony during a single day and estimated the number of bats roosting in a cluster, then the number of clusters on each major branch, and continuing this way until all roost trees of the colony were covered. New observers initially estimated colony size in parallel with trained observers, thus ensuring that the same technique was followed and data remained comparable. For previous counts and a detailed description of methods to estimate colony size see Hayman et al. [54].

### Meta-analysis of foraging distances of pteropodid bats

We compiled literature data on linear distances between day roosts and foraging areas as well as on body mass of Old Wold fruit bats (S4 Table and S1 Appendix) to contextualize the foraging distances of *E*. *helvum* in our study, and to assess the scaling of foraging distances with body size. These data were log-transformed and the relationship analysed with a linear regression (SigmaPlot 12).

## Results

### Seasonal population fluctuations

**Fig 1:**
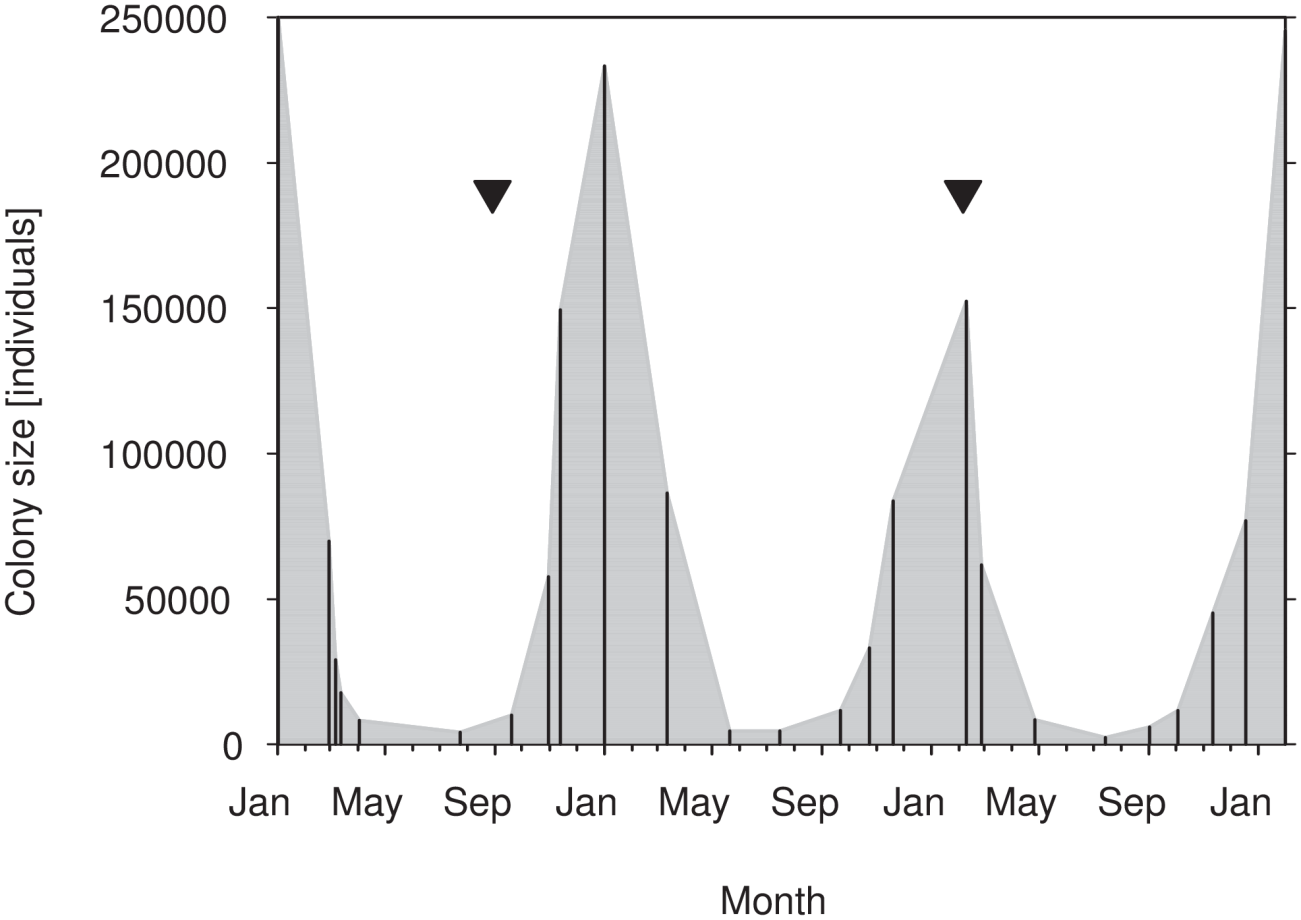
Seasonal colony fluctuations of *E*. *helvum* in Accra from January 2009 through January 2012. Triangles indicate tracking periods of the present study during population low (wet season 2009) and population high (dry season 2011); vertical lines represent colony counts.

Monitoring from January 2009 through January 2012 showed a cyclical fluctuation of colony size, with peak numbers during the dry season (October–March) and population minima during the wet season (April–September; Fig 1). Peak numbers (152,000–250,000 individuals) were ca. 50- up to 70-fold higher than the following minima (2,000–4,000 individuals).

### Tracking data

We downloaded complete wet season data from six of the 10 loggers that returned to the colony within the lifetime of the batteries. This covered 2.0–3.4 nights of tracking data per individual. One additional logger (#1082) downloaded a partial dataset, but for unknown reasons the battery failed after the data for the first 23 hours 23 min had downloaded. During the dry season 2011 we downloaded data from nine out of 20 loggers, which covered 1.0–6.0 nights of tracking data per individual.

### Seasonal differences in size of core and foraging areas as well as foraging distances

**Fig 2:**
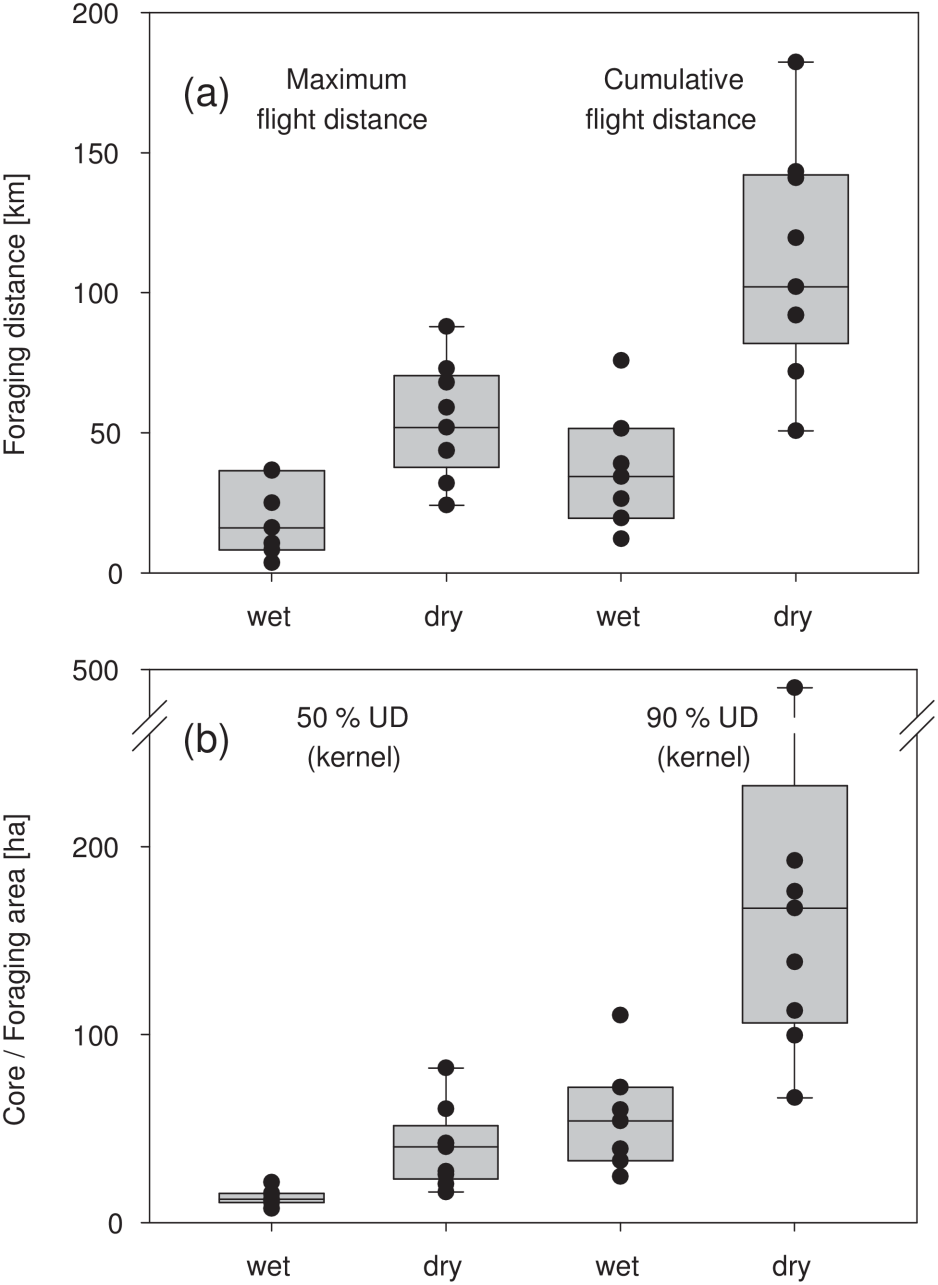
Seasonal changes of maximum and cumulative flight distances (a), and in size of core and foraging areas (b). Box plots show maximum flight distances from the colony to the most distant foraging area and the mean of daily cumulative flight distances. Box plots of core and foraging areas show the 50% and 90% kernel density UDs (Table 1). Dots represent raw data.

Both core and foraging areas as determined with kernel density estimation were significantly and on average about three-fold larger in the dry than in the wet season (Mann-Whitney U– 50% UD: p = 0.002; 90% UD: p = 0.003; 95% UD: p = 0.003; Table 1 and Fig 2). Although UDs of core and foraging areas calculated with the LoCoH-method showed the same trend (dry season areas were, on average, 4–13-fold larger), differences were not significant, which is probably explained by the large variance (Mann-Whitney U– 50% UD: p = 0.916; 90% UD: p = 0.290; 95% UD: p = 0.290; Table 1).

**Fig 3:**
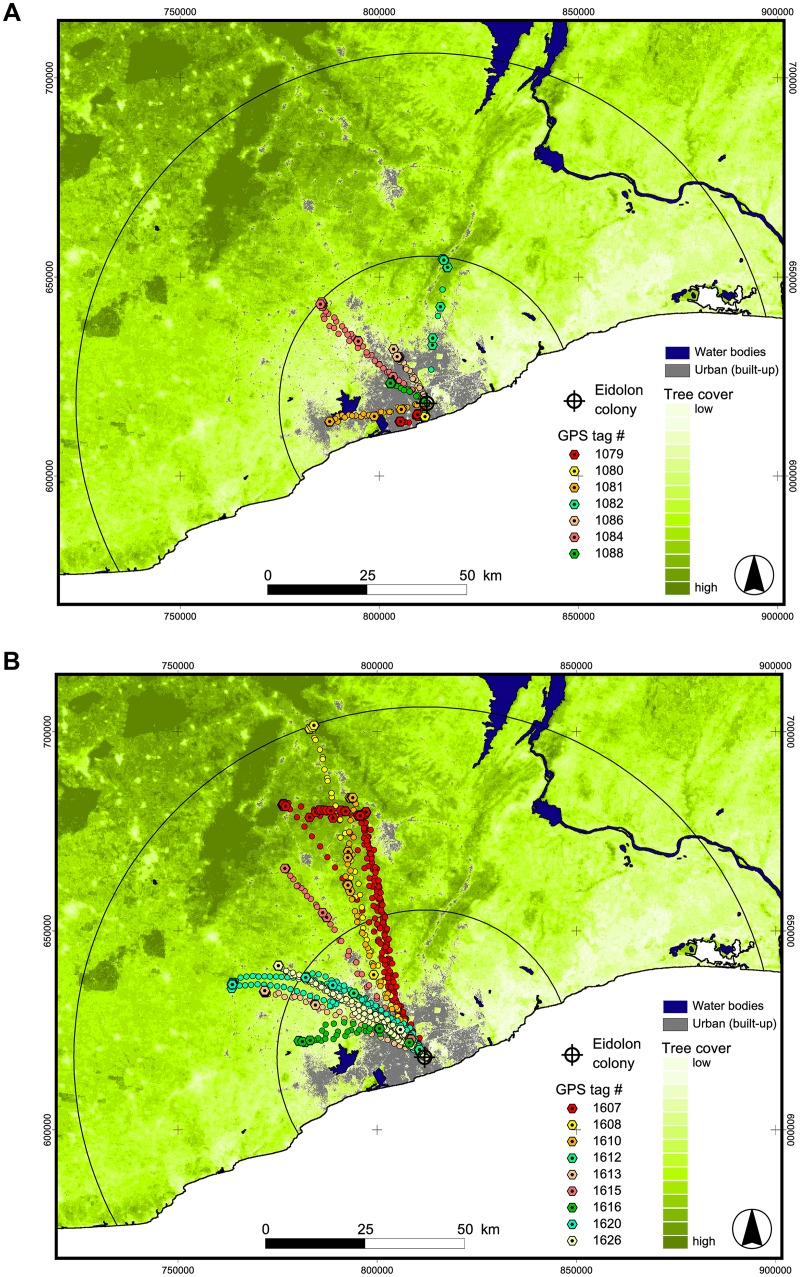
GPS tracks of *E*. *helvum* from wet (a) and dry season (b). Round dots represent commuting and roosting locations, and octagons foraging locations of *E*. *helvum*. Black circles indicate the maximum foraging distance of wet season (37 km) and dry season (88 km). Southern part of map corresponds to Atlantic Ocean. See Supporting Information (S3 Fig) for detailed maps of foraging areas of selected individuals.

The mean cumulative distance covered per night as well as the maximum distance between the colony and the respective foraging areas were significantly larger in the dry than in the wet season (Mann-Whitney U—mean cumulative distance/night: p = 0.003; maximum distance to foraging site: p = 0.006; Table 1, Figs 2 and 3). On average, these distances tripled during the dry season compared to the wet season.

### Habitat use in relation to season

**Fig 4:**
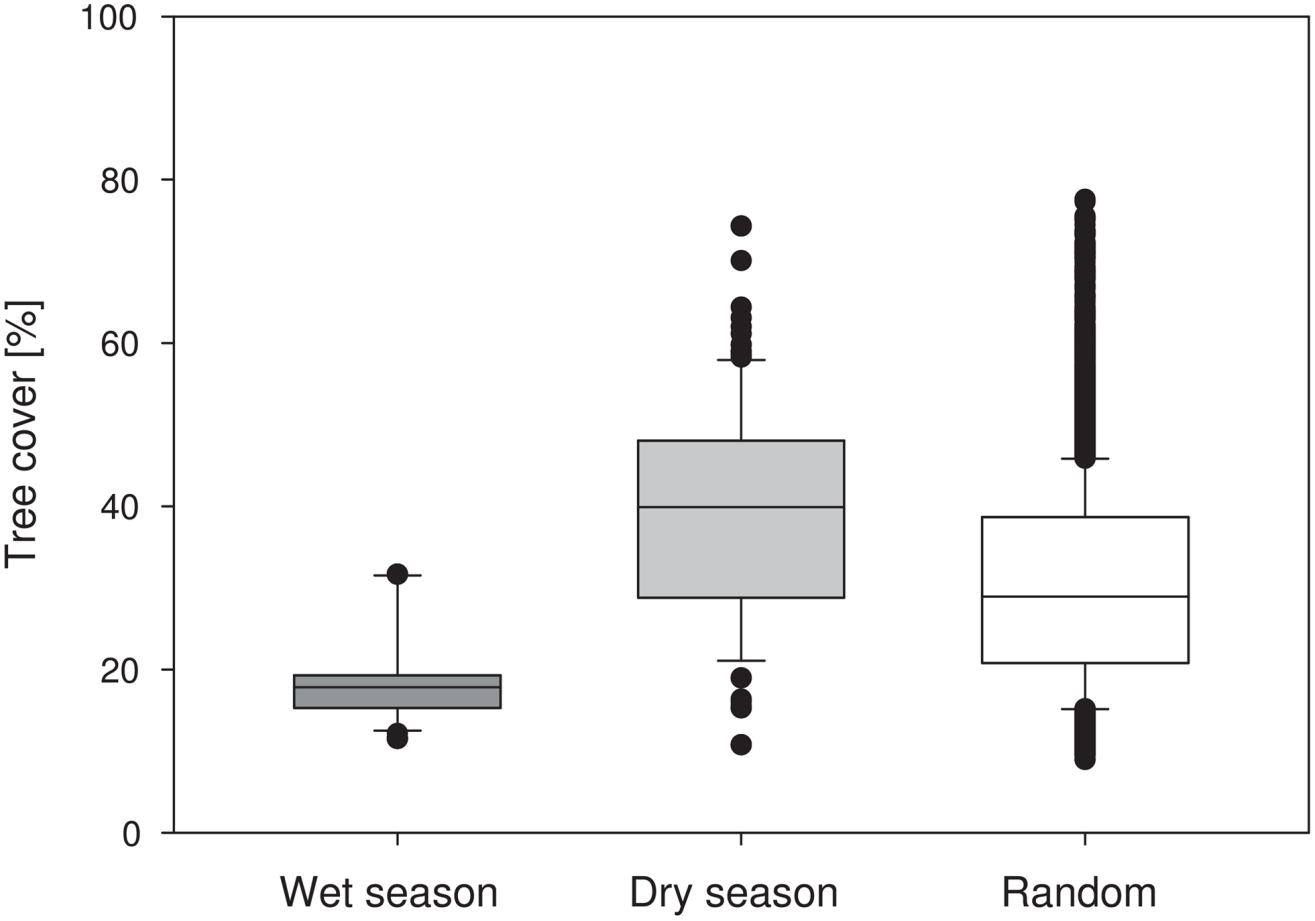
Habitat use of *E*. *helvum* with respect to tree cover. Box plot of foraging points during the wet (n = 306) and dry season (n = 225) compared to the frequency distribution of 10,000 random points within a radius of 88 km around the colony site. Black dots: outliers beyond the 5^th^ / 95^th^ percentiles.

During the wet season, bats used foraging areas with significantly lower tree cover, while during the dry season they used areas with significantly higher tree cover, compared to the random expectation (Fig 4; Kruskal-Wallis one-way ANOVA on ranks: H = 410.355, p < 0.001, all pair-wise comparisons significantly different from each other based on Dunn’s test).

Habitat use in relation to built-up areas (broadly corresponding to urban and suburban areas) revealed a parallel picture, with bats foraging 2–3 times more frequently in urban classes during the wet season while being found 1.5–5 times less frequently in these areas during the dry season compared to random expectations (see S2 Fig). This pattern held true irrespective of the spatial grain of the analysis.

These results were supported when based on mean values of each individual: in the wet season, bats used foraging areas with lower tree cover as well as urban space more frequently compared to the dry season (Mann-Whitney U—tree cover: p = 0.004; urban 4 m: p = 0.005, urban 100 m: p = 0.033; urban 232 m: p = 0.010; S3 Table).

### Behavioural data

We found no effect of foraging distance on activity budgets of the animals other than that of commuting flights (Table 2). Although cumulative and maximum foraging distances, and thus time spent flying, increased dramatically from wet to dry season, there was no significant difference between the seasons regarding time spent in foraging flight and/or resting/otherwise active. Bats spent more time moving (i.e., active but not flying) during the wet season (Table 2).

**Table 2:** Summary of activity budgets of tracked *E*. *helvum* during the wet vs. dry season based on acceleration data (wet: n = 6 individuals, dry: n = 9 individuals) .

		Wet season	Dry season	Mann-Whitney U	U'	p
Night	n° of acc. bursts	1587 ± 617	1505 ± 1281			
	range	780–2341	738–4729			
	% commuting flights	9.1 ± 6.1	26.1 ± 7.2	3	51	**0.0028**
	% foraging flights	3.7 ± 0.7	4.8 ± 2.5	12	42	0.0879
	% resting	34.0 ± 8.1	29.8 ± 13.5	24	30	0.7756
	% moving	52.0 ± 0.1	39.0 ± 0.1	10	53	**0.0132**
Day	n° of acc. bursts	1533 ± 655	1439 ± 1193			
	range	685–2876	694–4312			
	% resting	69.1 ± 12.1	71.7 ± 5.0			
24 h	% resting	51.0 ± 8.8	50.3 ± 6.6	26	28	0.9546

### Food types

Wet season: We visited the foraging areas of all bats and identified most of their food trees (Table 3). The most frequently used food tree was neem (*Azadirachta indica*), which was eaten by four of the seven bats. Other food plants were mango (*Mangifera indica*), papaya (*Carica papaya*), sea almond (*Terminalia catappa*)–all introduced and/or cultivated plants except two fig species (*Ficus thonningii* and *F*. *vallis-choudae*). In two cases it was not possible to identify the exact food tree. Both of these bats were foraging in gardens where they may have fed on banana (*Musa* sp.) and/or papaya. All bats but one foraged in the city or the suburbs of Accra, or in the 36 km distant town of Akwapim-Mampong (#1082). Bat #1086 left the urban environment to forage in a papaya plantation. One of the fig trees (*F*. *vallis-choudae*, bat # 1084) was in a rural landscape near a quarry north of Accra; however this bat’s main foraging tree was a neem tree in the middle of an urban environment. In one case (#1079) we were not able to find a fruit tree; however, there was a row of largely defoliated mahogany trees at the site (*Khaya senegalensis*). *Eidolon helvum* has been reported to feed on various other leaves as well as bark [55,56], so this individual may have been eating mahogany leaves.

**Table 3:** Tracking nights, flight distances, foraging habitat and food types utilized by tracked *E*. *helvum* during wet vs. dry season. Food plants in *italics* = introduced and/or cultivated, **bold** = native, (+1) if consumption of additional food plant uncertain.

	Bat #	# nights tracked	Mean cumulative distance/night [km]	Max. distance to foraging site [km]	Foraging habitat	Food plant	Food type	# foraging trees
Wet	1079	2.9	19.5	8.2	urban	*mango* (**mahogany**)	fruit (leaves?)	2
			19.1–19.9 (n = 2)					
	1080	2.0	12.1	3.5	urban	*neem*	fruit	1 (+1)
			11.1–13.1 (n = 2)					
	1081	3.0	51.5	24.9	urban	*neem*	fruit	1 (+1)
			50.2–52.3 (n =)					
	1082	1.0	38.9 (n = 1)	36.5	urban	**fig1** (*papaya*, *banana*)	fruit	2
	1084	3.4	34.3	16.1	urban	*neem*, **fig2** (**oil palm**, *banana*, *papaya*)	fruit	2
			33.9–35.0 (n = 3)					
	1086	2.0	75.7	36.7	plantation	*papaya*	fruit	1
			75.1–76.4 (n = 2)					
	1088	2.0	26.4	10.5	urban	*neem*, *sea almond*	fruit	3
			26.3–26.5 (n = 2)					
	Mean ± SD		36.9 ± 21.4	19.5 ± 13.5				
	Median		34.3	16.1				
Dry	1607	6.0	140.9	72.9	rural (2), urban	**kapok**, **African tulip**	flower	3
			128.1–165.2 (n = 6)					
	1608	1.0	182.3 (n = 1)	87.9	rural	n.d.	n.d.	2
	1610	1.0	143.2 (n = 1)	67.9	rural	**kapok** (**African tulip**)	flower	2
	1612	1.0	50.6 (n = 1)	24.1	rural (2), urban	**kapok**	flower	3
	1613	1.0	92.0 (n = 1)	43.6	rural	*cassia*	flower?, leaves?	2
	1615	1.0	119.5 (n = 1)	59.0	rural	**kapok**	flower	1
	1616	2.0	71.8	31.9	rural	**kapok**	flower	2
			70.9–72.6 (n = 2)					
	1620	2.0	102.1	51.9	rural	n.d.	n.d.	3
			75.0–129.3 (n = 2)					
	1626	2.0	91.9	43.5	rural	**kapok**	flower	1
			91.0–92.9 (n = 2)					
	Mean ± SD		110.5 ± 40.4	53.6 ± 20.4				
	Median		102.1	51.9				

Dry season: Food resources were more uniform as most bats fed almost exclusively on flowers (Table 3), in particular those of kapok trees (*Ceiba pentandra*). Two individuals also visited flowering African tulip (*Spathodea campanulata*) while GPS locations of another individual clustered around a grove of *Cassia* (*Senna*) *siamea* trees. Flowers of the latter species are unlikely to provide nectar to fruit bats [57], and this species has not previously been reported in the diet of *E*. *helvum*; however, the leaves are eaten by humans as well as by fruit bats in Asia [55], from where the tree originates. Most of the kapok and African tulip trees were in small groves or even single individuals left in clear-cut areas or cacao plantations, and the bats flew past fruiting trees of species they consumed during the wet season, especially neem.

### Scaling of foraging distances of Old World fruit bats in relation to body size

**Fig 5:**
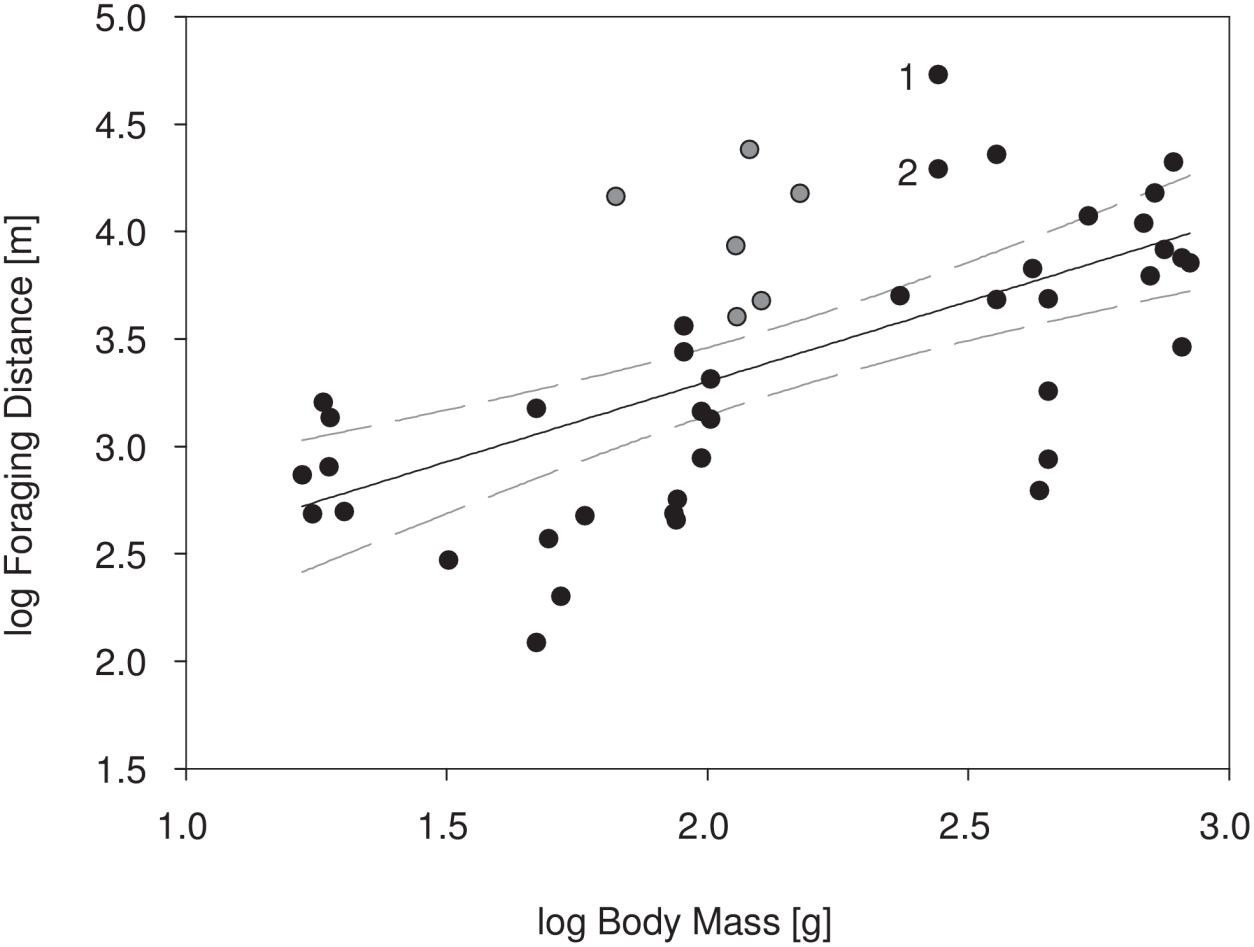
Foraging distances of Old World fruit bat species (Pteropodidae) in relation to body mass. 1: *E*. *helvum*, dry season, 2: *E*. *helvum*, wet season. Linear regression (y = 0.746x + 1.810, R^2^ = 0.384) with 95% confidence bands shown. Grey dots show cave-roosting *Rousettus* spp. See Supporting Information (S4 Table and S1 Appendix) for literature data.

Body size and foraging distance of Old World fruit bats are positively correlated (y = 0.746x + 1.810, R^2^ = 0.384, p < 0.0001, Fig 5). Based on the 95% confidence interval of the linear regression, highly colonial fruit bats including *E*. *helvum*, *Rousettus aegyptiacus*, *R*. *madagascariensis*, *R*. *leschenaultii* and *Pteropus tonganus* forage farther from the colony than other pteropodid bats.

## Discussion

The colony size of *Eidolon helvum* in Accra fluctuated dramatically and in a temporally consistent pattern over three years, with colony maxima of several hundred thousand individuals during the late dry season (January–February) and minima of a few thousand individuals during the wet season (May–September). The pattern agrees with the hypothesis that straw-coloured fruit bats reside in colonies along the West African coast during the dry season, and then migrate into northern savannas with the onset of the wet season, following concomitant resource flushes [12].

Our tracking studies were scheduled to coincide with the seasonal population minima and maxima. The concurrent increase of commuting distances, cumulative flight distances, foraging areas and flight time with population size, and especially the magnitude of change in these parameters, is intriguing (Figs 2 and 3, Tables 1 and 3). It is tempting to speculate that intraspecific competition increased strongly at peak population size during the dry season. According to optimal foraging theory, individuals should minimize travel distance, time and energy expenditure to locate food patches while maximizing food intake at these patches [58]. In a central-place forager such as *E*. *helvum*, it is likely that exploitation of food resources in the immediate vicinity of the colony is intense, and that the density of foraging bats decreases with increasing distance from the colony. In consequence, a substantial proportion of the colony might be forced to forage at much larger distances during the population high, leading to the observed increase in movement parameters. Similar patterns have been documented in central-place foraging seabirds where colony size was positively correlated with foraging distance and energy expenditure used for foraging [17,59,60]. Interestingly and in contrast to expectations, the high-resolution activity budgets of tracked bats did not reflect a correlated increase of energetic costs incurred by longer commuting flights during the dry season. If commuting flights were very costly, we would have expected the bats to spend more time foraging at the feeding sites, and / or reduced activity in the colony during the day. Neither of these reactions was apparent (Table 2). Activity budgets may not be the right measure to reveal such potential trade-offs, but the high resolution of the acceleration data should at least indicate such a trend, if present, as other studies have shown [61,62].

Alternatively or additionally, some of the differences between dry and wet season may be due to seasonal changes in the resource landscape, and potentially unrelated to colony size. Our diet data show a clear shift from introduced and cultivated fruit trees used during the wet season (in particular neem) to flower resources (mostly kapok trees) during the dry season. If kapok trees are, on average, found further from the colony than the wet season fruit resources, this could lead to the observed increase in foraging distances. However, neem as a steady-state fruiting tree was available also during the dry season but not used at all by our tracked individuals. Perhaps some types of fruit provide fall back staple food, but are less preferred in spite of greater spatial proximity and higher density, or other food sources become necessary as seasonal needs of the bats change. The latter effect, if true, should be more evident in females with increased energetic demands during reproduction (pregnancy during the dry season, see [13]), but this explanation seems unlikely as we only tracked males.

Currently, our data are temporal snapshots from the extremes in colony size. Critical tests to distinguish between these alternatives will require longer tracking periods, preferably covering an entire year. This should reveal whether movement parameters closely match the change in colony size and thus reflect competition, if they mirror the seasonal distribution of fruit and flower resources, or both. Given that bats showed rather stereotypic use of foraging areas over the course of several nights, medium- to long-term tracking of bats will reveal whether bats sequentially switch from one set of foraging areas to the next, both in terms of seasonal changes in resource availability but possibly also related to local depletions of resources over time. All of these avenues critically depend on the development of new solar-powered loggers that allow tracking of bats over longer time spans than in the present study.

The seasonal changes not only affected movement parameters but also habitat use. During the wet season, *E*. *helvum* foraged in patches characterized by comparatively low tree cover in (sub-) urban areas. Dry season foraging areas had higher tree cover and were mostly located beyond the (sub-) urban periphery (Figs 3 and 4). We cannot determine yet if this shift in habitat use is a consequence of the spatial distribution of these factors and correlated travel distance, i.e. tree cover increasing and urban areas decreasing with distance from the colony in the centre of Accra. Interestingly almost all of the plants consumed during the wet season were human-cultivated, introduced and/or invasive (see Table 3). It remains unclear if the observed partial migration is a recent phenomenon, but the urban landscape may offer predictable resource availability that the bats can fall back or even exclusively feed on to bridge periods of food paucity, which may become more severe with increased land use and climate change.

### Foraging distances of Old World fruit bats in relation to body mass

The foraging distances covered by *E*. *helvum* during the dry season by far surpass any previously recorded flight distance of bats between day roosts and foraging sites (S4 Table). Theory would predict a positive relationship between body mass and daily travel distance [63,64]. Indeed, currently available data on Old World fruit bats suggest such a relationship, though explaining a rather small amount of variation across species (R^2^ = 0.38, Fig 5). Apparently factors other than body size are important in determining this aspect of space use in pteropodid bats. Interestingly, all species with exceptionally large foraging distances (*E*. *helvum*, *Rousettus aegyptiacus*, *R*. *madagascariensis*, *R*. *leschenaultii*, and *Pteropus tonganus*; S4 Table) are highly gregarious central-place foragers, and both *E*. *helvum* and the three *Rousettus* species also show high fidelity to their day roosts, exploiting the resource landscape over longer time periods rather than shifting colonies in a nomadic fashion. Disentangling the various factors that influence foraging distance of fruit bats will be a major step forward to predict their ecological role as seed dispersers and pollinators. Our data clearly show that compared to other Old World fruit bats, *E*. *helvum* is likely to provide exceptional long-distance services in this regard, especially for pollen carried on the fur and small seeds ingested and defecated during flight. These predictions are based on daily distances travelled and do not even include potential dispersal extremes that could be achieved during migration.

## Conclusion and Outlook

Accra is one of the fastest growing cities in West Africa [65]. Currently both dry and wet season populations of straw-coloured bats seem to have sufficient food resources within accessible distances from the central colony in the downtown area of the city. In fact, the suburbs with plenty of introduced and cultivated fruit trees might provide an increased food supply, and seed dispersal of neem trees by the bats could have created a positive feed-back loop in this regard, i.e. bats planting their own food resources [66–68]. Several *Pteropus* species in Australia and Japan seem to have likewise profited from introduced and cultivated food resources in (sub-) urban environments [69–73]. In addition, the spatio-temporal resource availability might have significant implications for the migratory pattern of *E*. *helvum* because an increased year-round food supply could lead to a higher proportion of resident individuals, thus shifting the proportion of migratory vs. non-migratory bats. However, increasing agglomeration of buildings and infrastructure might eventually result in a decrease of food resources in distances energetically worthwhile. Unless the colony in Accra is persecuted with massive force, we do not expect *E*. *helvum* to disappear from this urban landscape, but it remains to be seen whether urban sprawl might eventually push resources so far from the colony that commuting flights are energetically too expensive to support the population sizes that are currently observed.

## Supporting Information

S1 Appendix Literature used to compile S4 Table. (PDF)

S1 Fig Examples of acceleration data. (PDF)

S2 Fig Habitat use of *Eidolon helvum* with respect to built-up areas. (PDF)

S3 Fig Examples of space use of *Eidolon helvum*. (PDF)

S1 Table Logger programming and measurements of tracked individuals. (PDF)

S2 Table Additional information on food trees utilized by *Eidolon helvum*. (PDF)

S3 Table Habitat utilization of tracked *Eidolon helvum* in relation to tree cover and built-up areas. (PDF)

S4 Table Literature data on foraging distance and body mass of Old World fruit bats used in Fig 5. (XLS)
