# Supplementary material for: Pronounced Seasonal Changes in the Movement Ecology of a Highly Gregarious Central-Place Forager, the African Straw-Coloured Fruit Bat (Eidolon helvum)
Source: PLoS One. 2015 Oct 14;10(10):e0138985. doi: 10.1371/journal.pone.0138985 (PMC4605647; doi:10.1371/journal.pone.0138985)
Supplement: S1 Appendix — (PDF) [file pone.0138985.s001.pdf]

Explanation of S4 Table

BM: body mass [g]; mean distance: mean distance b/w roost and foraging site(s) [km]; error type: SD – standard deviation, SE – standard error; max distance: maximum distance b/w roost and foraging site(s) [km]; range distance: range of distances b/w roost and foraging site(s) [km]; midpoint range: arithmethic midpoint of range of distances [km]; long axis mean: mean distance of long axis of home range [km]; long axis range: range of distances of long axis of home range [km]; proxy: colour-coded value used in regression; sample size: number of tracked individuals; day roost inside / outside FA: whether day roosts were reported to be located inside or outside of foraging area(s).

References for tracking studies of Old World fruit bats (Pteropodidae) included in S4 Table

Andrianaivoarivelo RA, Ramilijaona OR, Racey PA, Razafindrakoto N, Jenkins RKB (2011) Feeding ecology, habitat use and reproduction of *Rousettus madagascariensis* Grandidier, 1928 (Chiroptera: Pteropodidae) in eastern Madagascar. Mammalia 75: 69-78.

Banack SA, Grant GS (2002) Spatial and temporal movement patterns of the flying fox, *Pteropus tonganus*, in American Samoa. Journal of Wildlife Management 66: 1154-1163.

Barclay RMR, Jacobs DS (2011) Differences in the foraging behaviour of male and female Egyptian fruit bats (*Rousettus aegyptiacus*). Can J Zool 89: 466-473.

Bonaccorso FJ, Winkelmann JR, Byrnes DGP (2005) Home range, territoriality, and flight time budgets in the black-bellied fruit bat, *Melonycteris melanops* (Pteropodidae). J Mamm 86: 931-936.

Bonaccorso FJ, Winkelmann JR, Dumont ER, Thibault KM (2002) Home range of *Dobsonia minor* (Pteropodidae): A solitary, foliage-roosting fruit bat in Papua New Guinea. Biotropica 34: 127-135.

Bonaccorso FJ, Winkelmann JR, Todd CM, Miles, AC (2014) Foraging movements of epauletted fruit bats (Pteropodidae) in relation to the distribution of sycamore figs (Moraceae) in Kruger National Park, South Africa. Acta Chiropterologica 16: 41-52.

Bradbury JW (1977) Lek mating behavior in the hammer-headed bat. Z Tierpsychol 45: 225-255.

de Jong C, Field HE, Tagtag A, Hughes T, Dechmann DKN, et al. (2013) Foraging behaviour and landscape utilisation by the endangered golden-crowned flying fox (*Acerodon jubatus*), The Philippines. PLoS ONE 8: e79665.

Eby P (1991) Seasonal movements of grey-headed flying-foxes, *Pteropus poliocephalus* (Chiroptera: Pteropodidae), from two maternity camps in northern New South Wales. Wildlife Research 18: 547-559.

Fenton MB, Brigham RM, Mills AM, Rautenbach IL (1985) The roosting and foraging areas of *Epomophorus wahlbergi* (Pteropodidae) and *Scotophilus viridis* (Vespertilionidae) in Kruger National Park, South Africa. J Mamm 66: 461-468.

Funakoshi K, Zubaid A (1997) Behavioural and reproductive ecology of the dog-faced fruit bats, *Cynopterus brachyotis* and *C. horsfieldi*, in a Malaysian rainforest. Mammal Study 22: 95-108.

Hodgkison R, Balding ST, Zubaid A, Kunz TH (2003) Roosting ecology and social organization of the spotted-winged fruit bat, *Balionycteris maculata* (Chiroptera: Pteropodidae), in a Malaysian lowland dipterocarp forest. J Trop Ecol 19: 667-676.

Jacobsen NHG, Viljoen PC, Ferguson W (1986) Radio tracking of problem fruit bats (*Rousettus aegyptiacus*) in the Transvaal with notes on flight and energetics. Z Säugetierk 51: 205-208.

Law BS (1993) Roosting and foraging ecology of the Queensland blossom bat (*Syconycteris australis*) in north-eastern New South Wales: Flexibility in response to seasonal variation. Wildlife Research 20: 419-431.

Law BS, Lean M (1999) Common blossom bats (*Syconycteris australis*) as pollinators in fragmented Australian tropical rainforest. Biol Conservation 91: 201-212.

Markus N, Hall LS (2004) Foraging behaviour of the black flying-fox (*Pteropus alecto*) in the urban landscape of Brisbane, Queensland. Wildl Res 31: 345-355.

Mildenstein TL, Stier SC, Nuevo-Diego CE, Mills LS (2005) Habitat selection of endangered and endemic large flying-foxes in Subic Bay, Philippines. Biol Conservation 126: 93-102.

Nair NG, Elangovan V, Sripathi K, Marimuthu G, Subbaraj R (1999) Foraging behavior of the Indian short-nosed fruit bat *Cynopterus sphinx*. Z Säugetierk 64: 187-191.

Nakamoto A, Kinjo K, Izawa M (2012) Ranging patterns and habitat use of a solitary flying fox (*Pteropus dasymallus*) on Okinawa-jima Island, Japan. Acta Chiropterologica 14: 387-399.

Nelson SL (2003) Nutritional Ecology of Old-World Fruit Bats: A Test of the Calcium-Constraint Hypothesis. University of Florida: Ph.D. Thesis. xiii+140 p.

Oleksy R, Racey PA, Jones G (2015) High-resolution GPS tracking reveals habitat selection and the potential for long-distance seed dispersal by Madagascan flying foxes *Pteropus rufus*. Global Ecology and Conservation 3: 678-692.

Palmer C, Woinarski JCZ (1999) Seasonal roosts and foraging movements of the black flying fox (*Pteropus alecto*) in the Northern Territory: Resource tracking in a landscape mosaic. Wildlife Research 26: 823-838.

Reiter J, Curio E (2001) Home range, roost switching, and foraging area in a Philippine fruit bat (*Ptenochirus jagori*). Ecotropica 7: 109-113.

Richter HV, Cumming GS (2008) First application of satellite telemetry to track African straw-coloured fruit bat migration. J Zool (Lond) 275: 172-176.

Rollinson DP (2012) Seasonal Home Range and Foraging Movements of the Wahlberg's Epauletted Fruit Bat (*Epomophorus wahlbergi*) in an Urban Environment. University of KwaZulu-Natal, Pietermaritzburg: MSc Thesis. viii+84 p.

Rollinson DP, Coleman JC, Downs CT (2013) Seasonal differences in foraging dynamics, habitat use and home range size of Wahlberg's epauletted fruit bat in an urban environment. African Zoology 48: 340-350.

Spencer HJ, Fleming TH (1989) Roosting and foraging behaviour of the Queensland tube-nosed bat *Nyctimene robinsoni* (Pteropodidae): Preliminary radio-tracking observations. Australian Wildlife Research 16: 413-420.

Spencer HJ, Palmer C, Parry-Jones KA (1991) Movements of fruit-bats in eastern Australia determined by radio-tracking. Wildlife Research 18: 463-468.

Tang Z, Chen Z, Ma J, Zhu G, Ma X, et al. (2010) Foraging areas of *Rousettus leschenaulti* on the Hainan Island of China. Current Zoology 56: 479-484.

Tsoar A, Nathan R, Bartan Y, Vyssotski A, Dell'Omo G, et al. (2011) Large-scale navigational map in a mammal. Proc Nat Acad Sci USA 108: E718-E724.

Watzke S (2006) Ressourcennutzung und Paarungssystem der nektarivoren Flughundart *Macroglossus minimus* (Pteropodidae: Macroglossinae) in West-Malaysia. Ludwig-Maximilians-Universität München: Ph.D. Thesis. 186 p.

Weber N, Duengkae P, Fahr J, Dechmann DKN, Phengsakul P, et al. (2015) High-resolution GPS tracking of Lyle's flying fox between temples and orchards in central Thailand. Journal of Wildlife Management: 10.1002/jwmg.904.

Winkelmann JR, Bonaccorso FJ, Goedeke EE, Ballock LJ (2003) Home range and territoriality in the least blossom bat, *Macroglossus minimus*, in Papua New Guinea. J Mamm 84: 561-570.

Winkelmann JR, Bonaccorso FJ, Strickler TL (2000) Home range of the southern blossom bat, *Syconycteris australis*, in Papua New Guinea. J Mamm 81: 408-414.

References for body masses of bats in S4 Table, if not given in tracking studies

Heideman PD, Heaney LR (1989) Population biology of fruit bats (Pteropodidae) in Philippine submontane rainforest. J Zool (Lond) 218: 565-586.

Miller CA, Wilson DE (1997) *Pteropus tonganus*. Mammalian Species (552): 1-6.

Monadjem A, Taylor PJ, Cotterill FPD, Schoeman MC (2010) Bats of Southern and Central Africa. A Biogeographic and Taxonomic Synthesis. Johannesburg: Wits University Press. xii+596 p.

Riek A, Körtner G, Geiser F (2010) Thermobiology, energetics and activity patterns of the eastern tube-nosed bat (*Nyctimene robinsoni*) in the Australian tropics: Effect of temperature and lunar cycle. J Exp Biol 213: 2557-2564.

Roberts BJ, Catterall CP, Eby P, Kanowski J (2012) Long-distance and frequent movements of the flying-fox *Pteropus poliocephalus*: Implications for management. PLoS ONE 7: e42532.
