# Supplementary material for: Pronounced Seasonal Changes in the Movement Ecology of a Highly Gregarious Central-Place Forager, the African Straw-Coloured Fruit Bat (Eidolon helvum)
Source: PLoS One. 2015 Oct 14;10(10):e0138985. doi: 10.1371/journal.pone.0138985 (PMC4605647; doi:10.1371/journal.pone.0138985)

Figure S1: Examples of acceleration data. Shown are four 15 s-bursts (recorded once a minute continuously). X-axis: time, y-axis: acceleration (m/s^2). a) flying – magnified inset shows oscillating acceleration on z-axis, which corresponds to wing beats; b) resting; c) moving; d) moving, specifically scratching; note that orientation (i.e. which axis is shifted by 1 g) is different from a) because bat is hanging upside down as opposed to horizontally flying.

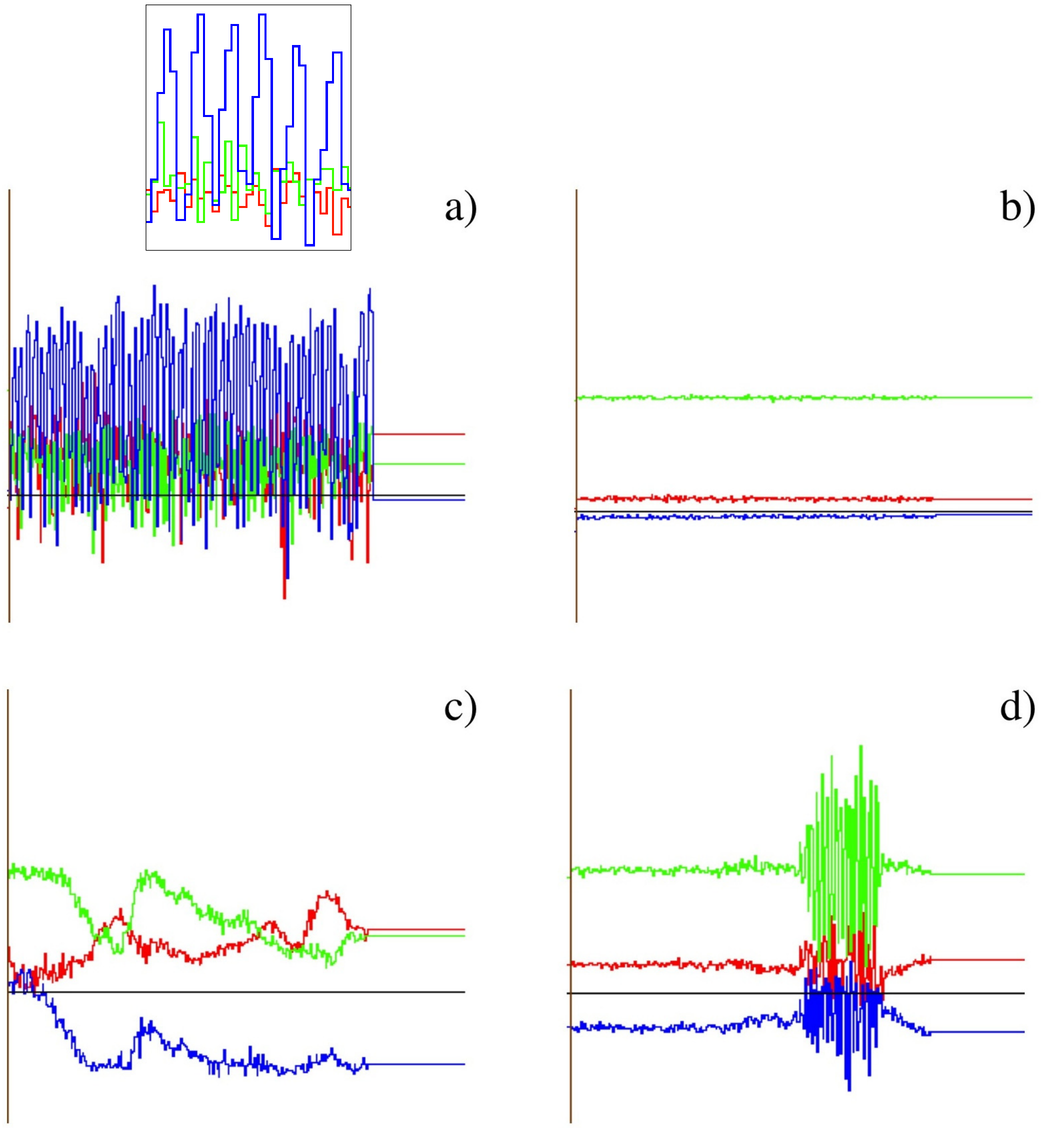

Supplement: S1 Fig — (PDF) [file pone.0138985.s002.pdf]
