# Supplementary material for: Pronounced Seasonal Changes in the Movement Ecology of a Highly Gregarious Central-Place Forager, the African Straw-Coloured Fruit Bat (Eidolon helvum)
Source: PLoS One. 2015 Oct 14;10(10):e0138985. doi: 10.1371/journal.pone.0138985 (PMC4605647; doi:10.1371/journal.pone.0138985)

Figure S2: Habitat use of *Eidolon helvum* with respect to built-up areas. Frequency distribution of foraging points during the wet season (n=357) and the dry season (n=225) compared to the frequency distribution of 10,000 random points within a radius of 88 km radius around the colony site. Percent urban is the proportion of points classified as “built up” out of the entire pool of points at three spatial grains (see Methods).

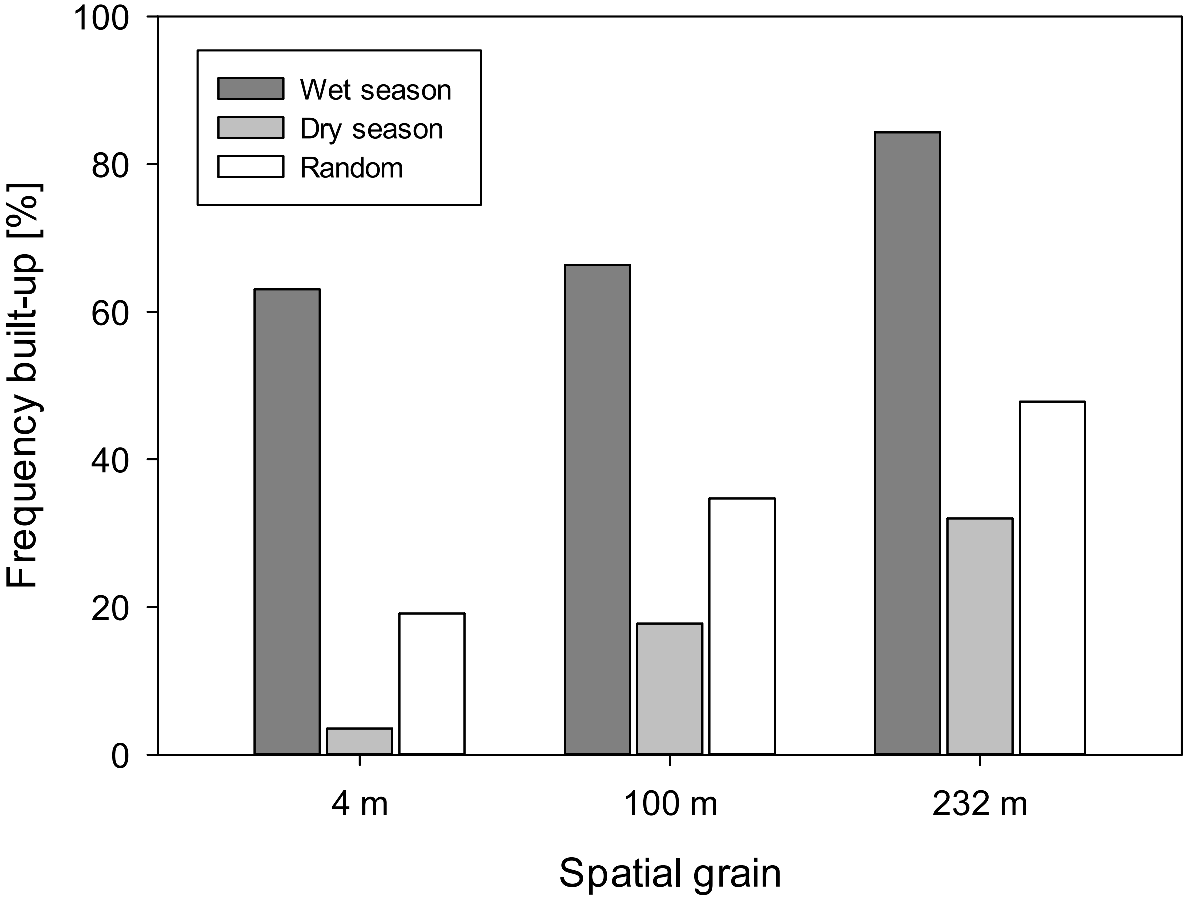

Supplement: S2 Fig — (PDF) [file pone.0138985.s003.pdf]
