# Supplementary material for: Pronounced Seasonal Changes in the Movement Ecology of a Highly Gregarious Central-Place Forager, the African Straw-Coloured Fruit Bat (Eidolon helvum)
Source: PLoS One. 2015 Oct 14;10(10):e0138985. doi: 10.1371/journal.pone.0138985 (PMC4605647; doi:10.1371/journal.pone.0138985)

Figure S3: Examples of space use of *Eidolon helvum*. Yellow dots: GPS-points, pale blue: foraging areas (95 % UD), dark blue: core areas (50 % UD). Map data © 2013 Google and DigitalGlobe / GeoEye.

Wet season (note that foraging sites are mostly located in (sub-) urban areas)

Bat #1088:

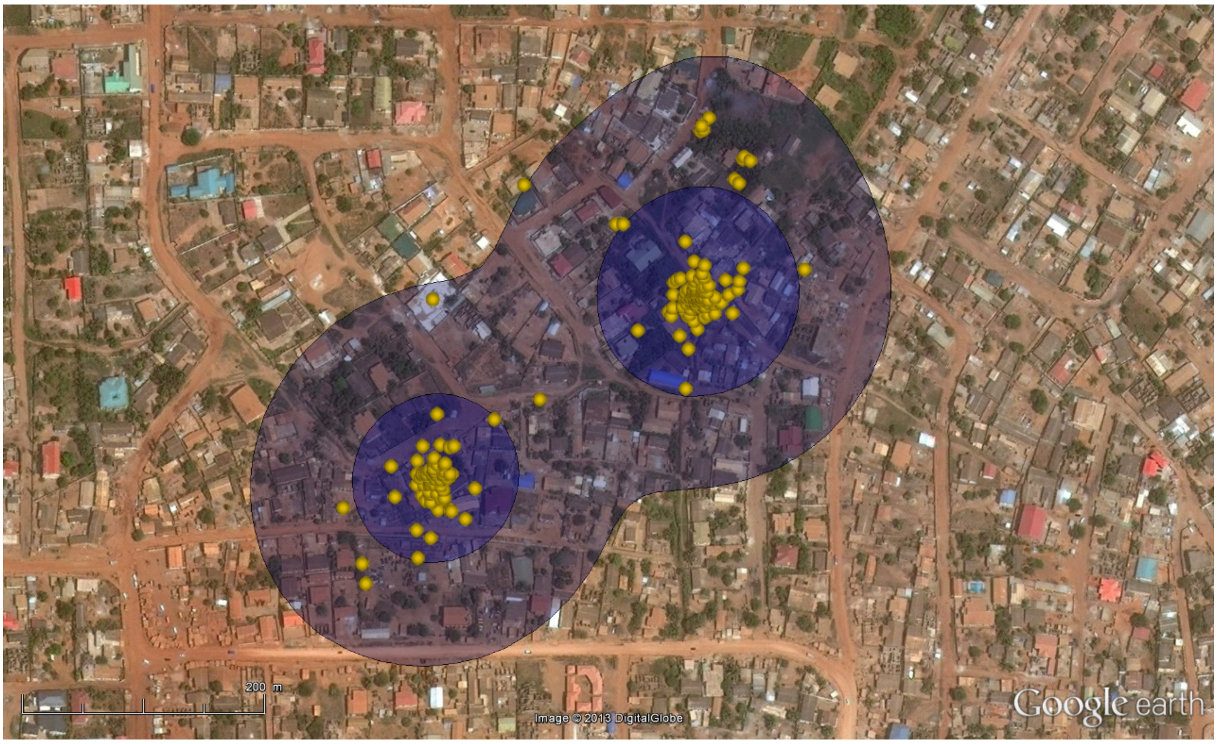

Bat #1078 overview:

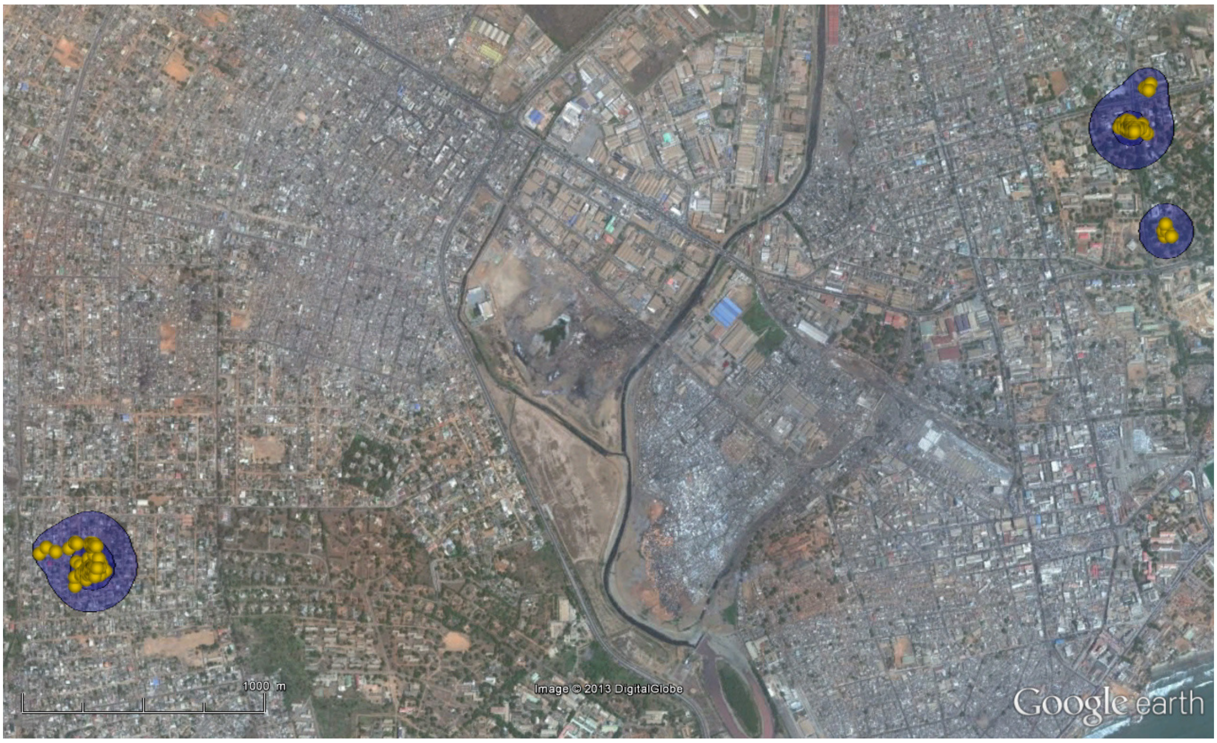

Bat #1078 detail:

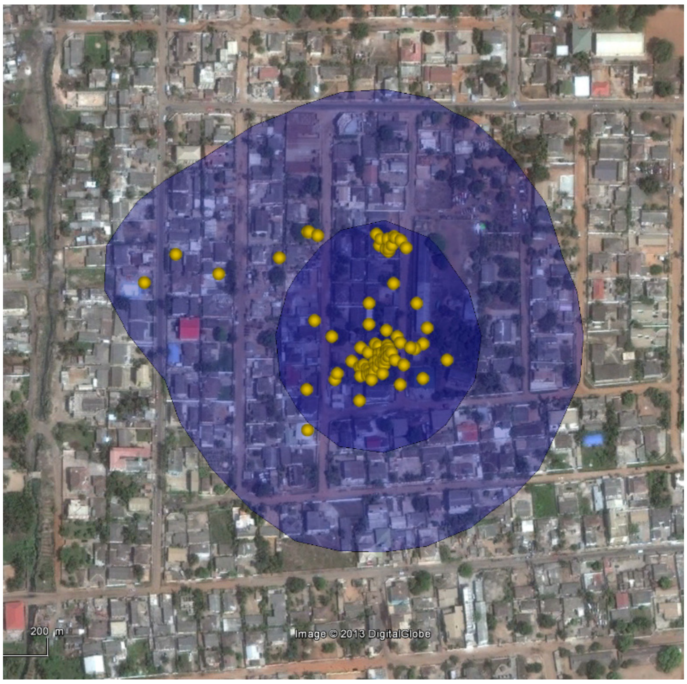

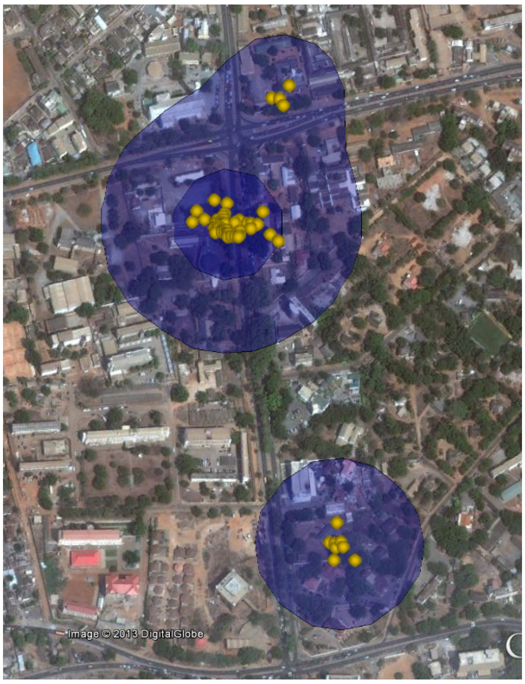

Bat #1080 (left) and #1081 (right):

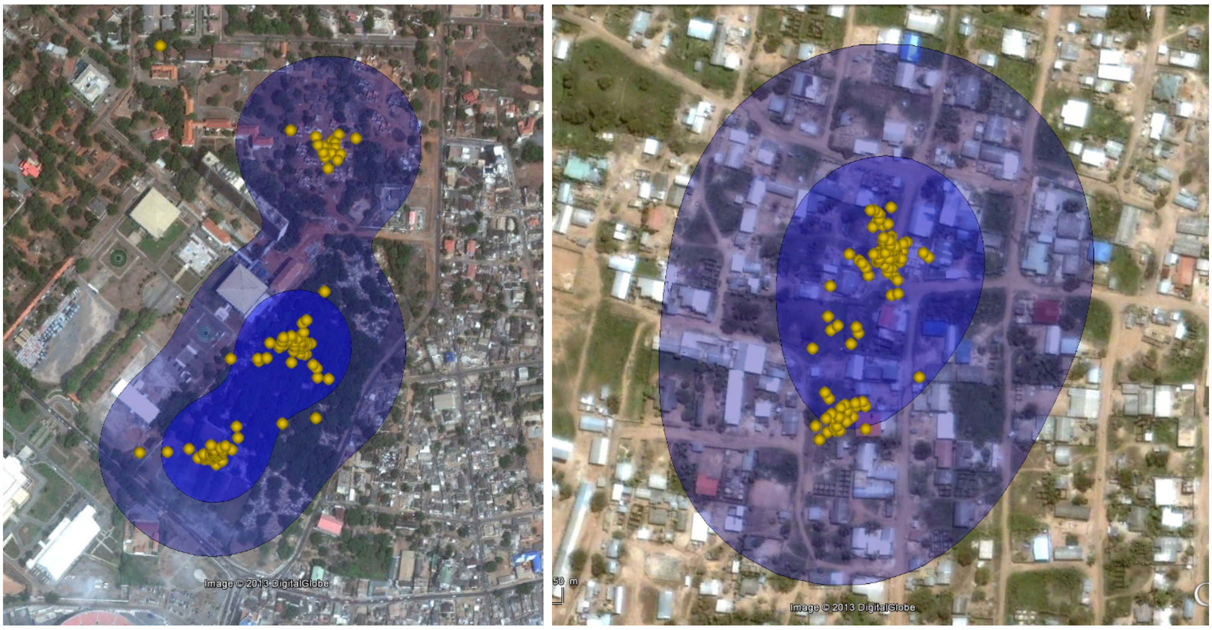

Bat #1084 overview (left) and detail (right):

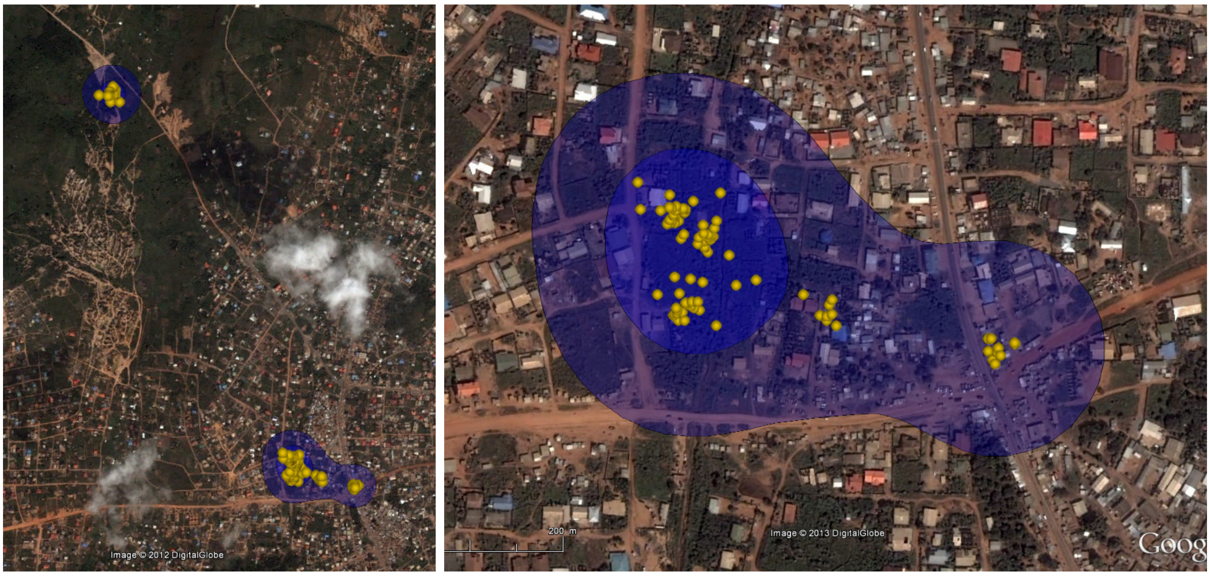

Bat #1078 overview:

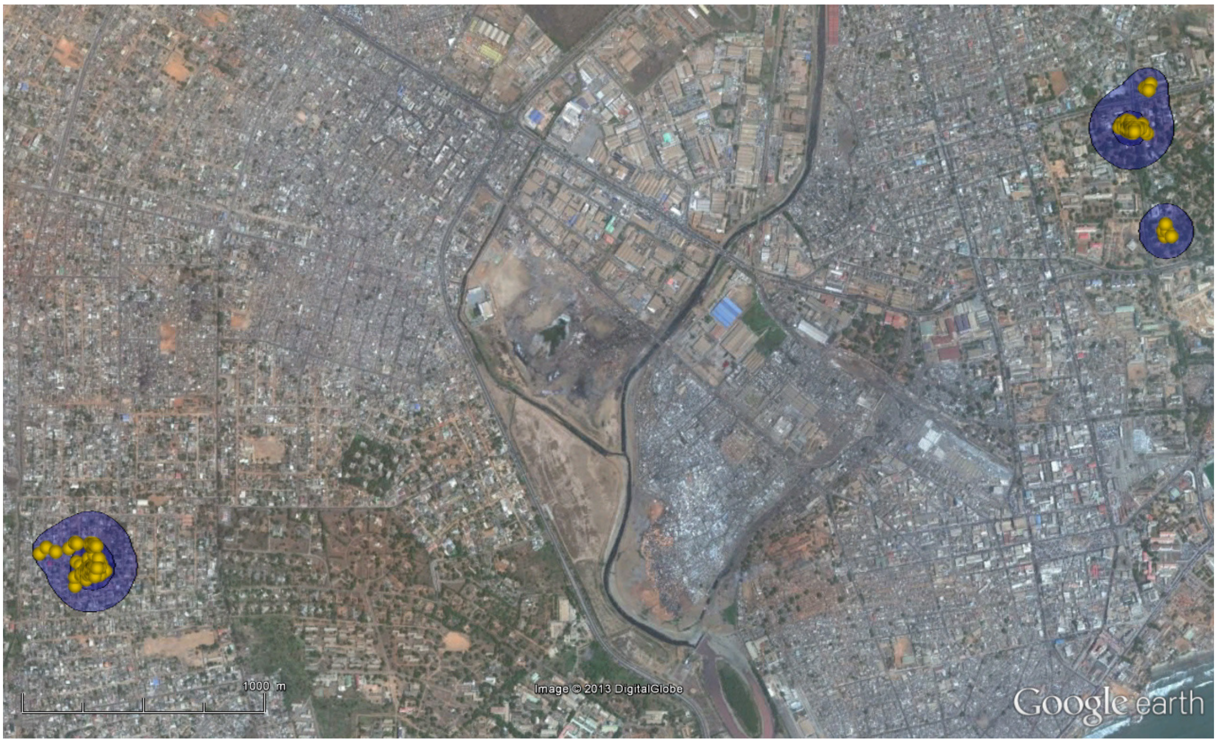

Bat #1078 detail:

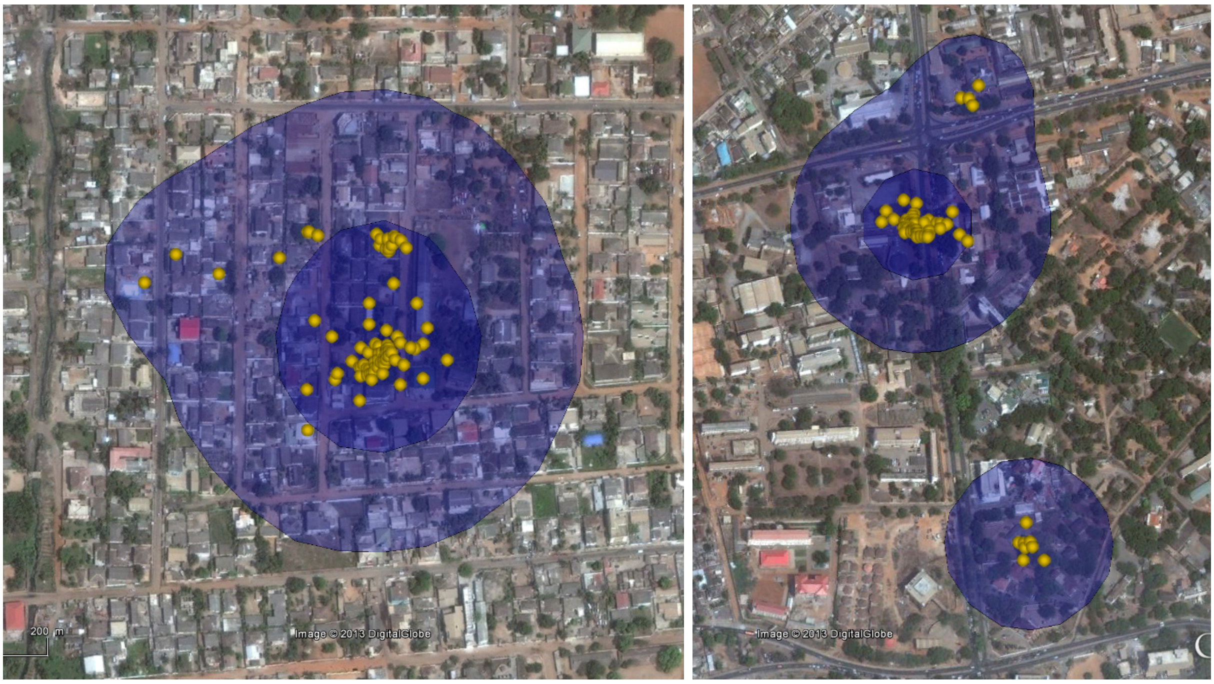

Bat #1620 overview:

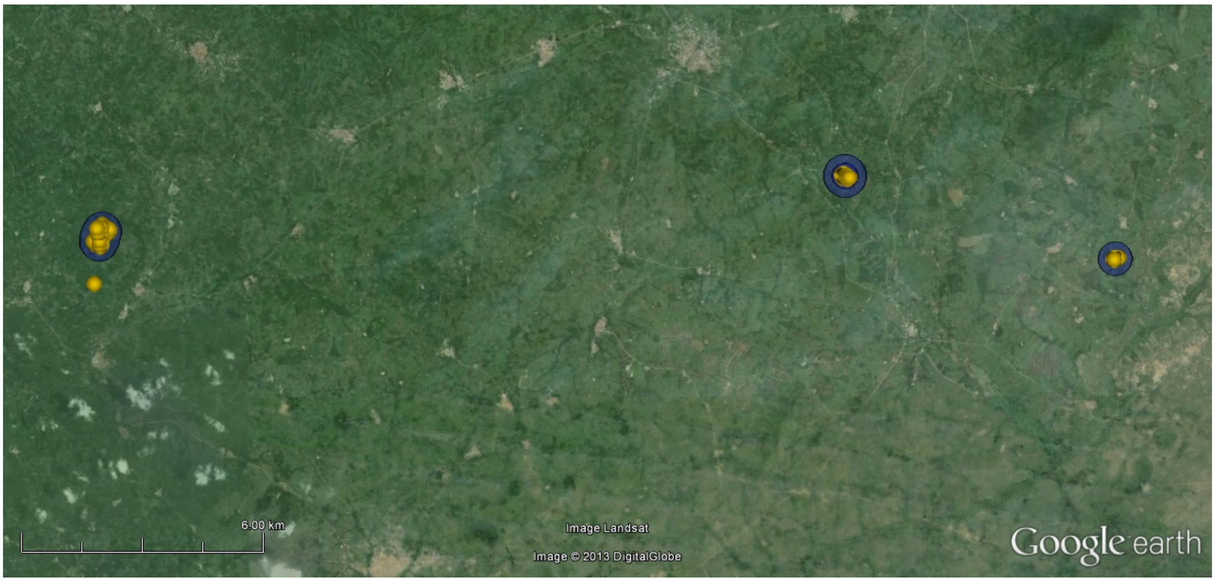

Bat #1620 detail (left: west, right: east):

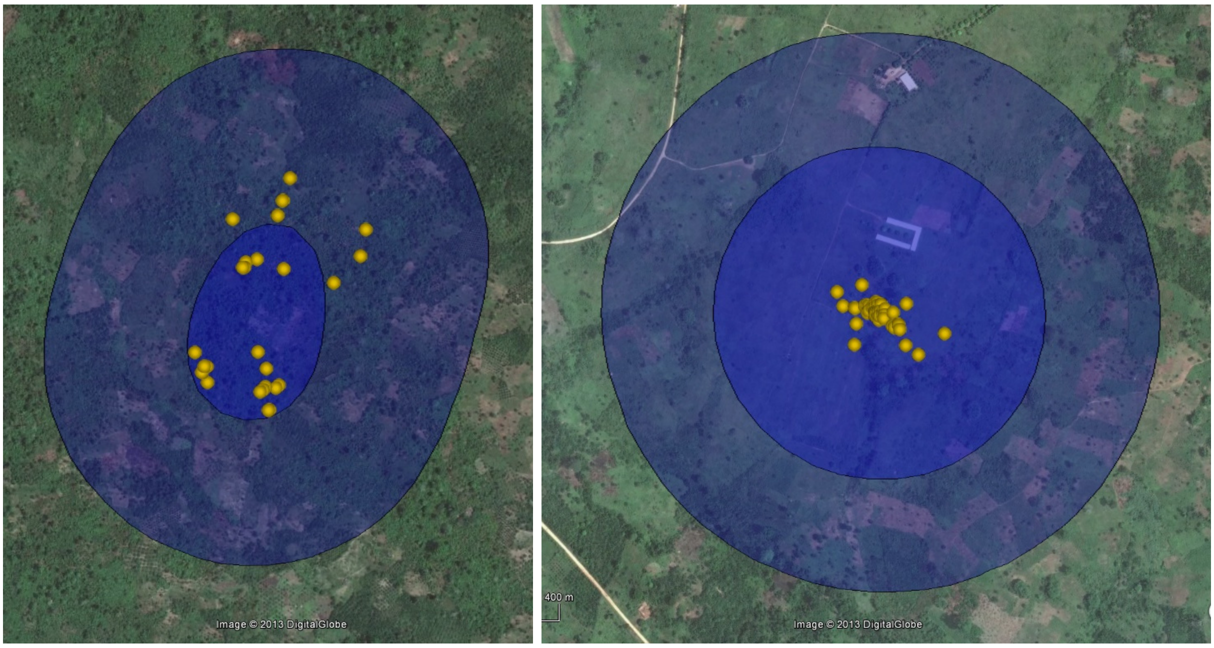

Bat #1626:

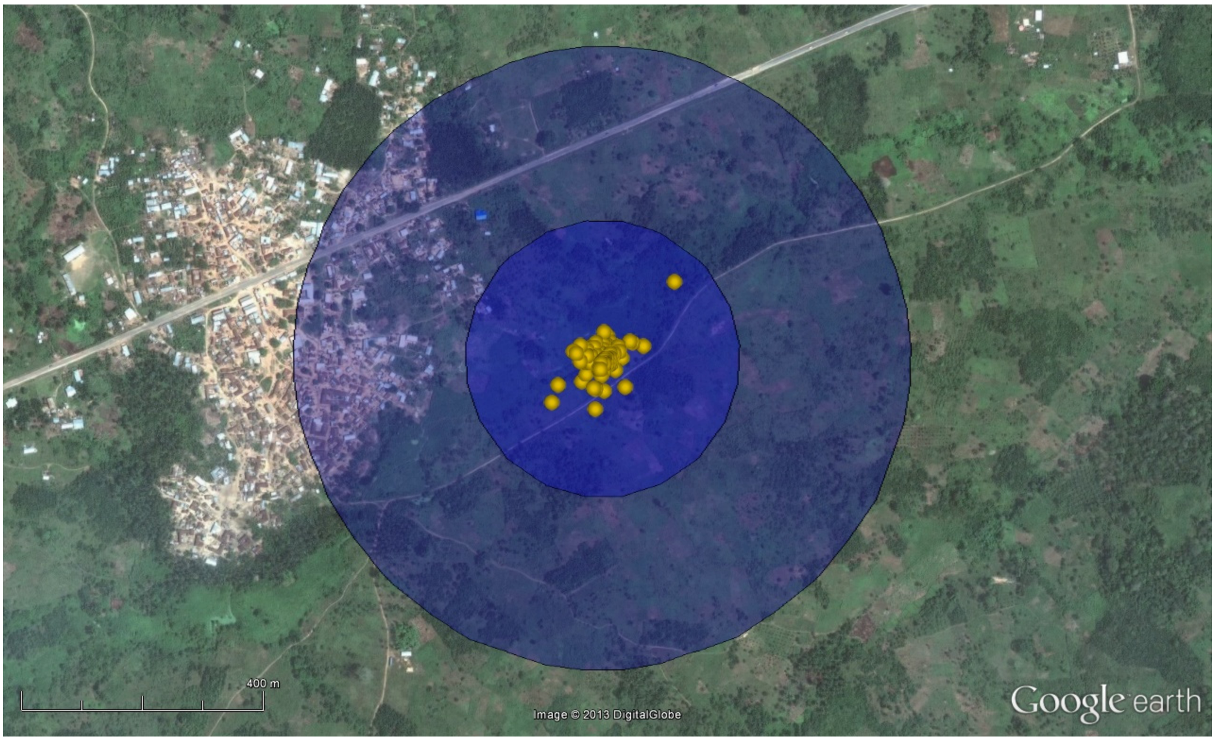

Supplement: S3 Fig — (PDF) [file pone.0138985.s004.pdf]
