# Supplementary material for: Pronounced Seasonal Changes in the Movement Ecology of a Highly Gregarious Central-Place Forager, the African Straw-Coloured Fruit Bat (Eidolon helvum)
Source: PLoS One. 2015 Oct 14;10(10):e0138985. doi: 10.1371/journal.pone.0138985 (PMC4605647; doi:10.1371/journal.pone.0138985)
Supplement: S1 Table — (PDF) [file pone.0138985.s005.pdf]

S1 Table: Logger programming and measurements of tracked individuals.

		Logger = bat ID	GPS interval [s]	Logger mass [g]	Bat mass [g]	Forearm length [mm]
Wet season	Cohort 1	1079	600	19.5	284	118.1
		1080	600	19.5	244	113.9
		1081	600	19.5	274	123.5
		1082	600	19.5	246	120.0
	Cohort 2	1084	900/300	19.5	239	115.1
		1086	900/300	19.5	277	118.2
		1088	300	19.5	247	120.0
Dry season	Cohort 3	1607	1800/300	24.0	321	124.7
		1608	1800/300	24.0	300	122.1
		1610	1800/300	24.0	270	114.7
		1612	1800/300	24.0	310	118.6
		1613	1800/300	24.0	305	123.4
		1615	1800/300	24.0	300	123.9
		1616	1800/300	24.0	292	121.0
		1620	1800/300	20.0	255	119.7
		1626	1800/300	20.0	280	119.1
