# Supplementary material for: Pronounced Seasonal Changes in the Movement Ecology of a Highly Gregarious Central-Place Forager, the African Straw-Coloured Fruit Bat (Eidolon helvum)
Source: PLoS One. 2015 Oct 14;10(10):e0138985. doi: 10.1371/journal.pone.0138985 (PMC4605647; doi:10.1371/journal.pone.0138985)
Supplement: S2 Table — (PDF) [file pone.0138985.s006.pdf]

S2 Table: Additional information on food trees utilized by *Eidolon helvum*.

Common name	Scientific name	Family	Eaten by bat #	Food type	Season eaten	Origin	Introduction to West Africa	Source for introduction	Previously reported in diet of *E. helvum*
Neem	*Azadirachta indica*	Meliaceae	1080, 1081, 1084, 1088	fruit	wet	Asia	1920ies (Accra Plains: 1950ies)	[1]	[2–6]
Mango	*Mangifera indica*	Anacardiaceae	1079	fruit	wet	Asia	18-19th century		[5,7–12]
Sea almond	*Terminalia catappa*	Combretaceae	1088	fruit	wet	West-Pacific	unknown	[13]	[7]
Banana	*Musa* sp.	Musaceae	(1082), (1084)	fruit	wet	Asia	BC		[8,11,14]
Papaya	*Carica papaya*	Caricaceae	(1082), (1084), 1086	fruit	wet	America	16-17th century	[15]	[4,8,10,12]
African mahogany	*Khaya senegalensis*	Meliaceae	(1079)	leaves?	wet	native			
Strangler fig	*Ficus thonningii*	Moraceae	1082	fruit	wet	native			[12]
Fig	*Ficus vallis-choudae*	Moraceae	1084	fruit	wet	native			[12]
Silk cotton tree	*Ceiba pentandra*	Malvaceae	1607, 1610, 1612, 1615, 1616, 1626	nectar	dry	native			[8,9,16–18]
Cassia	*Cassia* (*Senna*) *siamea*	Fabaceae	1613	blossoms? leaves?	dry	Asia			
African tulip	*Spathodea campanulata*	Bignoniaceae	1607, (1615)	nectar	dry	native			

References

1. Chamberlain JR, Childs FJ, Harris PJC (2000) An Introduction to Neem, its Use and Genetic Improvement: Centre for Natural Resources and Development (CNRD), University of Oxford, UK. 52 p.
2. Kock D (1969) Die Fledermaus-Fauna des Sudan (Mammalia, Chiroptera). Abh Senckenb Naturforsch Ges 521: 1-238.
3. Kock D (1978) Vergleichende Untersuchung einiger Säugetiere im südlichen Niger (Mammalia: Insectivora, Chiroptera, Lagomorpha, Rodentia). Senckenbergiana biol 58: 113-136.
4. Ayensu ES (1974) Plant and bat interactions in West Africa. Ann Missouri Bot Gard 61: 702-722.
5. Koch-Weser S (1984) Fledermäuse aus Obervolta, W-Afrika (Mammalia: Chiroptera). Senckenbergiana biol 64: 255-311.
6. Schmutterer H (1992) Beobachtungen über Nutznießer der Früchte des Niembaumes (*Azadirachta indica*) in Afrika mit besonderer Berücksichtigung von Senegal. Anzeiger für Schädlingskunde, Pflanzenschutz, Umweltschutz 65: 1-4.
7. Jones C (1972) Comparative ecology of three pteropid bats in Rio Muni, West Africa. J Zool (Lond) 167: 353-370.
8. Funmilayo O (1979) Ecology of the straw-coloured fruit bat in Nigeria. Rev Zool Afr 93: 589-600.
9. Thomas DW (1982) The Ecology of an African Savanna Fruit Bat Community: Resource Partitioning and Role in Seed Dispersal. University of Aberdeen: Ph.D. Thesis. vii+206 p.
10. Niamien CJM, Yaokokoré-Béibro HK, Koné I, Yao S, N'Goran KE (2009) Données préliminaires sur les habitudes alimentaires des chauves-souris paillées, *Eidolon helvum* (Kerr, 1792) (Chiroptera: Pteropodidae) de la commune d'Abidjan plateau (Côte d'Ivoire). Agronomie Africaine 21: 231-240.
11. Richter HV, Cumming GS (2006) Food availability and annual migration of the straw-colored fruit bat (*Eidolon helvum*). J Zool (Lond) 268: 35-44.
12. Webala PW, Musila S, Makau R (2012) Population Ecology, Diet and Movement of Straw-coloured Fruit Bats (*Eidolon helvum*), Western Kenya: Final Report to Rufford Small Grants Foundation. 23 p. Available at <http://www.rufford.org/files/10078-1%20Detailed%20Final%20Report_0.pdf>
13. Morton JF (1985) Indian almond (*Terminalia catappa*), salt-tolerant, useful, tropical tree with "nut" worthy of improvement. Economic Botany 39: 101-112.
14. Osmaston HA (1965) Pollen and seed dispersal in *Chlorophora excelsa* and other Moraceae, and in *Parkia filicoidea* (Mimosaceae), with special reference to the role of the fruit bat, *Eidolon helvum*. Commonwealth Forestry Review 44: 97-105.
15. Alpern SB (1992) The European introduction of crops into West Africa in precolonial times. History in Africa 19: 13-43.
16. Baker HG, Harris BJ (1959) Bat-pollination of the silk-cotton tree, *Ceiba pentandra* (L.) Gaertn. (sensu lato), in Ghana. J West Afr Sci Ass 5: 1-9.
17. Harris BJ, Baker HG (1959) Pollination of flowers by bats in Ghana. Nigerian Field 24: 151-159.
18. Pettersson S (2005) Bats and Bat Flowers in a West African Rainforest Community. Göteborg University: Ph.D. Thesis.
