# Supplementary material for: Pronounced Seasonal Changes in the Movement Ecology of a Highly Gregarious Central-Place Forager, the African Straw-Coloured Fruit Bat (Eidolon helvum)
Source: PLoS One. 2015 Oct 14;10(10):e0138985. doi: 10.1371/journal.pone.0138985 (PMC4605647; doi:10.1371/journal.pone.0138985)
Supplement: S3 Table — (PDF) [file pone.0138985.s007.pdf]

S3 Table: Habitat utilization of tracked *E. helvum* in relation to tree cover (mean grid values of foraging points) and built-up areas ('urban').

	Bat #	Tree cover [%]	Urban 4 m	Urban 100 m	Urban 232 m	Sample size
Random		30.1	19.1%	34.7%	47.9%	10,000
Wet	1079	14.3	99.3%	100.0%	100.0%	141
	1080	18.0	17.8%	27.7%	100.0%	101
	1081	17.8	97.6%	100.0%	100.0%	126
	1082	30.7	59.6%	76.6%	78.7%	47
	1084	18.4	70.0%	70.0%	97.5%	120
	1086	30.2	2.0%	2.0%	11.8%	51
	1086	16.6	93.6%	100.0%	100.0%	202
Dry	1607	50.8	0.0%	15.1%	37.6%	186
	1608	57.1	0.0%	0.0%	3.7%	27
	1610	50.2	0.0%	3.3%	13.3%	30
	1612	22.9	4.0%	32.0%	32.0%	25
	1613	38.4	0.0%	0.0%	35.9%	39
	1615	40.4	10.3%	86.2%	96.6%	29
	1616	23.7	25.3%	28.0%	72.0%	75
	1620	36.3	0.0%	0.0%	0.0%	90
	1626	35.5	4.9%	4.9%	4.9%	164

For urban, the percentage of foraging points in this class is given (binary data) while for tree cover the mean value of all grid cells is shown (continuous data). Also shown are the respective values for 10,000 random points.
